# Supplementary material for: Effectiveness of COVID-19 vaccines against severe COVID-19 among patients with cancer in Catalonia, Spain
Source: Nat Commun. 2024 Jun 19;15:5088. doi: 10.1038/s41467-024-49285-y (PMC11187152; doi:10.1038/s41467-024-49285-y)
Supplement: Supplementary file 1 — Supplementary Information [file 41467_2024_49285_MOESM1_ESM.pdf]

Effectiveness of COVID-19 Vaccines Against Severe COVID-19 Among Patients with Cancer in Catalonia, Spain

SUPPLEMENTARY MATERIAL

FIGURE 1. COVID-19 cases and Vaccine Rollout in Catalonia ....p. 2
FIGURE 2. Vaccines Products Types by Time and Age ....p. 3
FIGURE 3. Flow-chart Primary Vaccination Scheme (Cohort A) ....p. 4
FIGURE 4. Flow chart Booster Vaccination Scheme (Cohort B)...................................p. 5
FIGURE 5. Primary Outcome Curves Days 0 to 30 after Vaccination...........................p. 6
FIGURE 6. Subgroup Analysis of Primary Outcome (Cohort A) ....p. 7
FIGURE 7. Subgroup Analysis of Primary Outcome (Cohort B) ....p. 8
FIGURE 8. Negative Outcomes HR Forest Plot..p. 9
FIGURE 9. Negative Outcomes Funnel Plot ....p. 10
FIGURE 10. Cumulative Hazard Curves Original and Restricted Matching (A)............p. 11
FIGURE 11. Cumulative Hazard Curves Original and Restricted Matching (B)............p. 12
FIGURE 12. Sensitivity Analysis Cohort A ....p. 13
FIGURE 13. Sensitivity Analysis Cohort B ....p. 14
FIGURE 14. Rolling Entry Matching (REM) Scheme ....p. 15
TABLE 1. Matched vs Unmatched (Cohort A) ....pp. 16-17
TABLE 2. Matched vs Unmatched (Cohort B) ....pp. 18-19
TABLE 3. Competing Risk Model ....p. 20
TABLE 4. Vaccine Effectiveness after Negative Outcomes Calibration ....p. 21
TABLE 5. Non-COVID-19 Outcomes (Cohort A) ....p. 22
TABLE 6. Non-COVID-19 Outcomes (Cohort B) ....p. 23
TABLE 7. Proportion of Deaths and Preceding Hospitalizations (Cohort A and B) ......p. 24
TABLE 8. Health Services Utilization after Vaccination (Cohort A and B)....................p. 25-26
TABLE 9. Restrictive Matching Baseline (Primary Vaccination)...................................p. 27-28
TABLE 10. Restrictive Matching Baseline (Booster Vaccination).................................p. 29-30
TABLE 11. Non-COVID-19 Outcomes (Restrictive Matching, Primary Vaccination)....p. 31
TABLE 12. Non-COVID-19 Outcomes (Restrictive Matching, Booster)........................p. 32
TABLE 13. COVID-19 Outcomes (Restrictive Matching)...p. 33
TABLE 14. Cohort Definitions (OMOP-CDM) ....p. 34
TABLE 15. Cancer Concept IDs (OMOP-CDM) ....p. 35-36
TABLE 16. Charlson Concept IDs (OMOP-CDM) ....p. 37
TABLE 17. Negative Outcomes Concept IDs (OMOP-CDM) ....p. 38

SUPPL. FIGURE 1. Absolute number of COVID-19 cases since the beginning of vaccination campaign in Spain (Figure A), weekly proportion of predominant variant of concern (VOC) by week (Figure B), and cumulative vaccine rollout for the first, second and third dose among patients with cancer (Figure C).

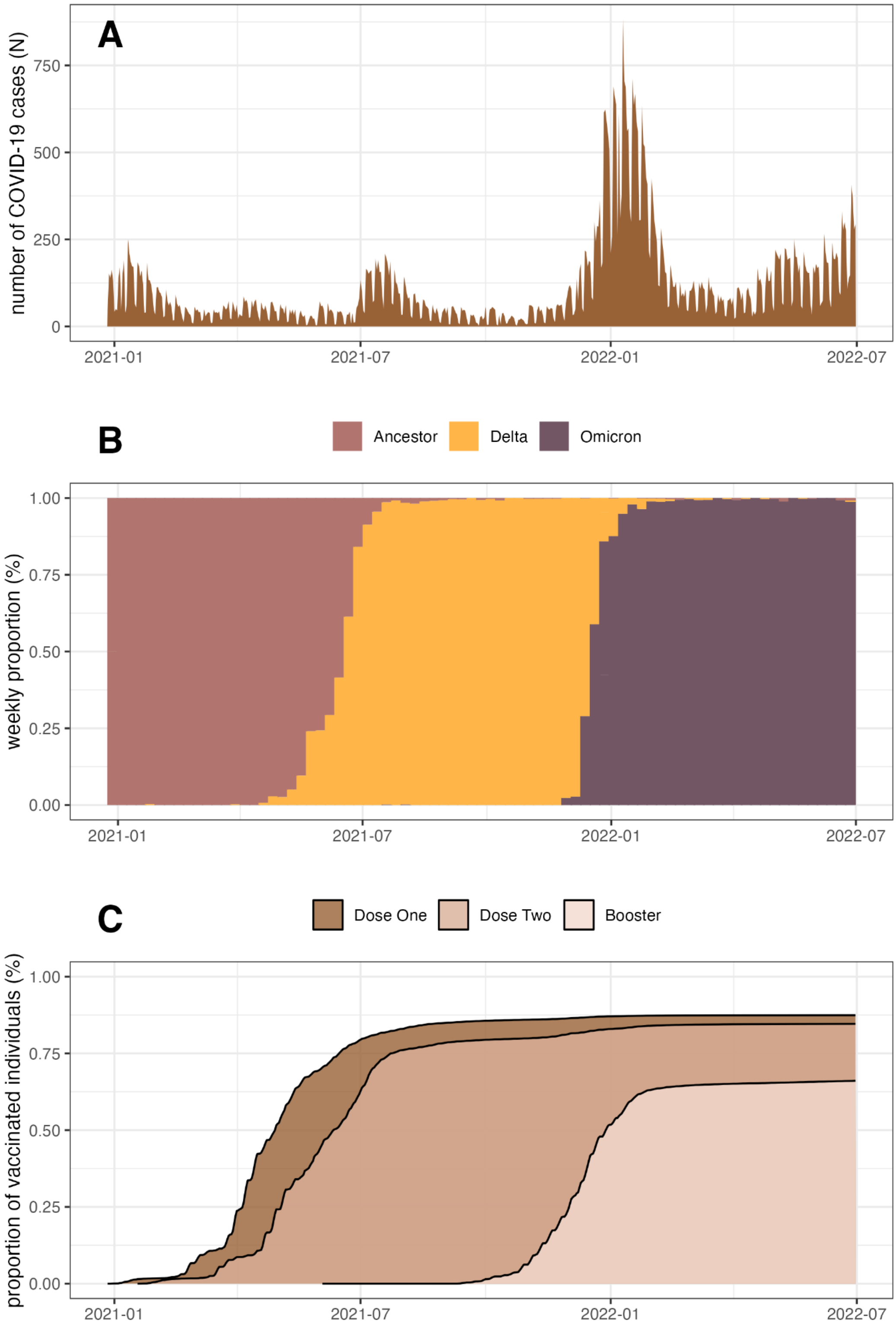

SUPPL. FIGURE 2. Absolute number of vaccines administered since the beginning of the vaccination campaign (27th December 2020) categorized by product type and age groups. Colors represent different vaccine products.

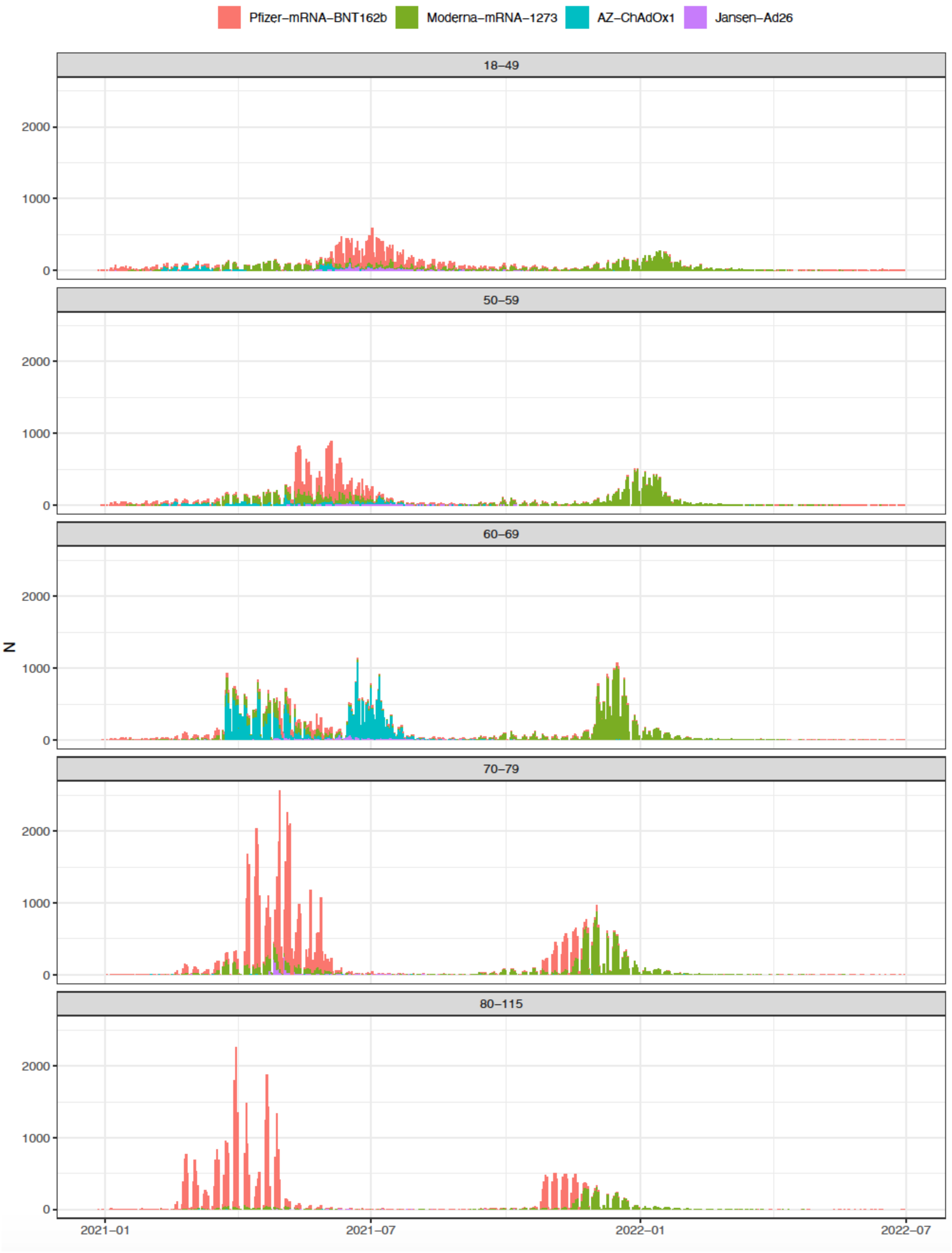

SUPPL. FIGURE 3. Flow-chart of participants included in the primary vaccination cohort (Cohort A) after the application of inclusion and exclusion criteria and matching. .

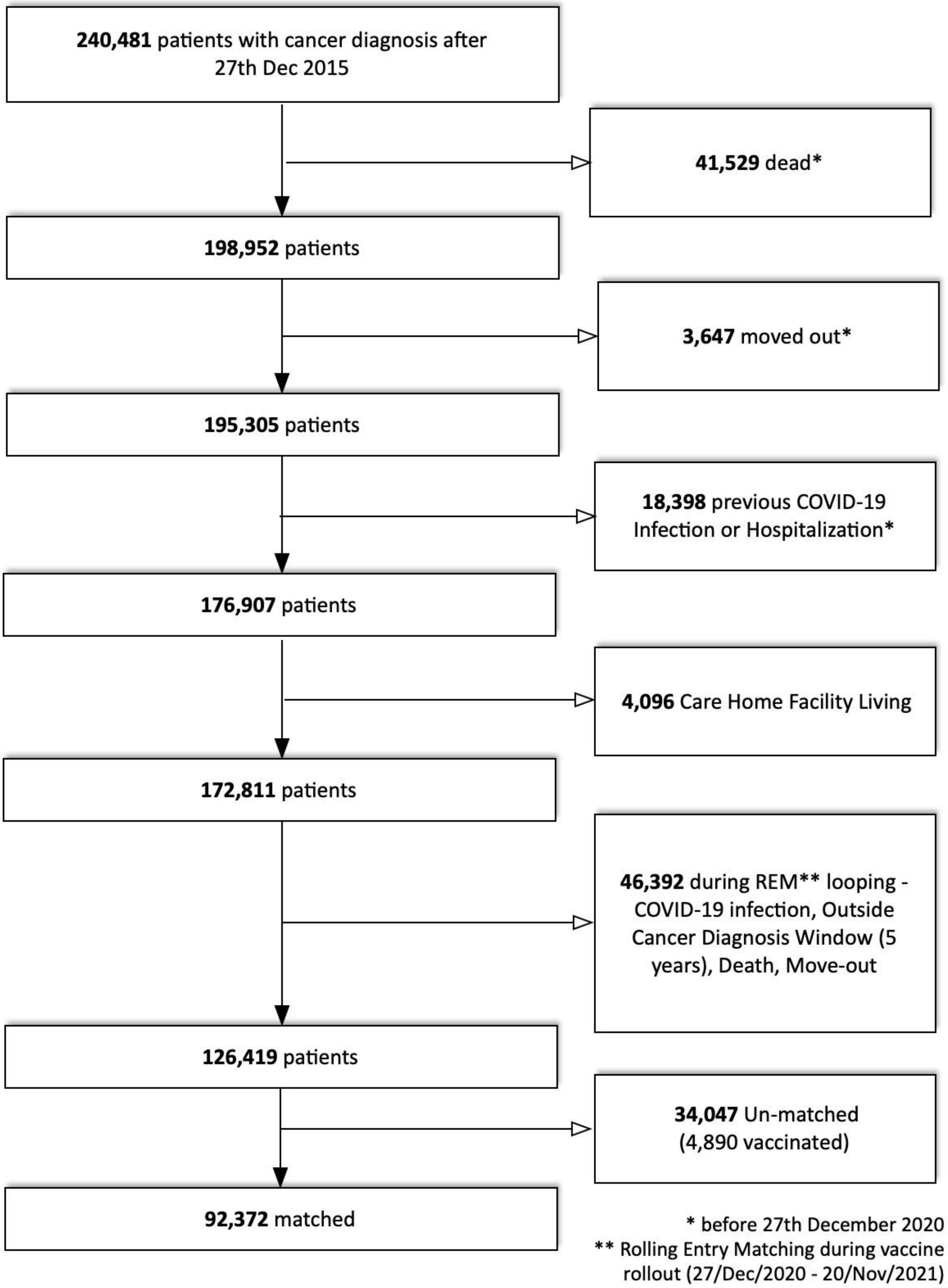

SUPPL. FIGURE 4. Flow-chart of participants included in the booster vaccination cohort (Cohort B) after the application of inclusion and exclusion criteria and matching.

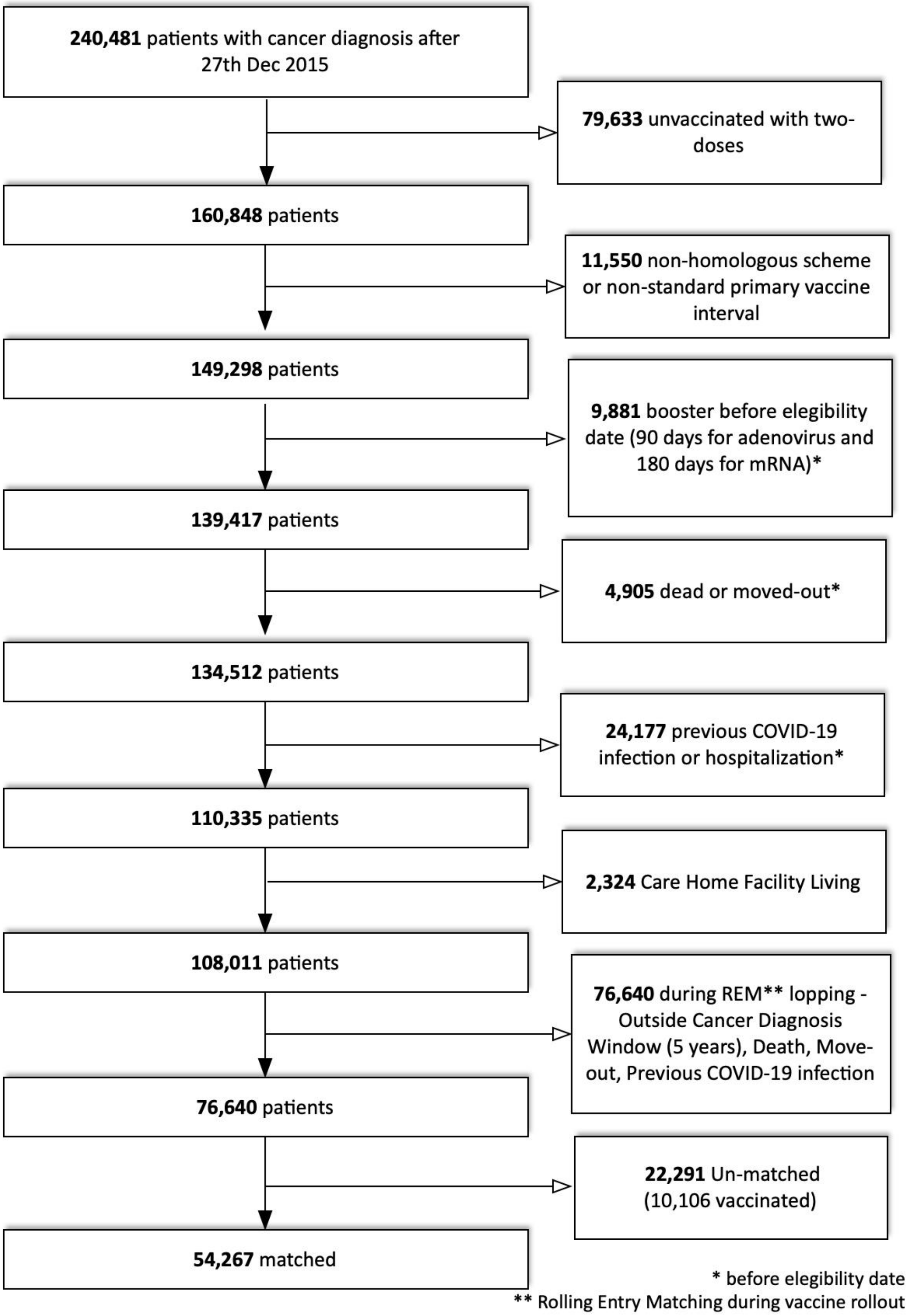

SUPPL. FIGURE 5. Cumulative incidence of the primary outcome (COVID-19 hospitalization) by treatment groups (vaccinated and controls) during the initial 30 days period for the primary vaccination (Figure A) and the booster vaccination (Figure B). The solid lines represent the estimated cumulative hazards, while the shaded areas indicate the 95% confidence intervals.

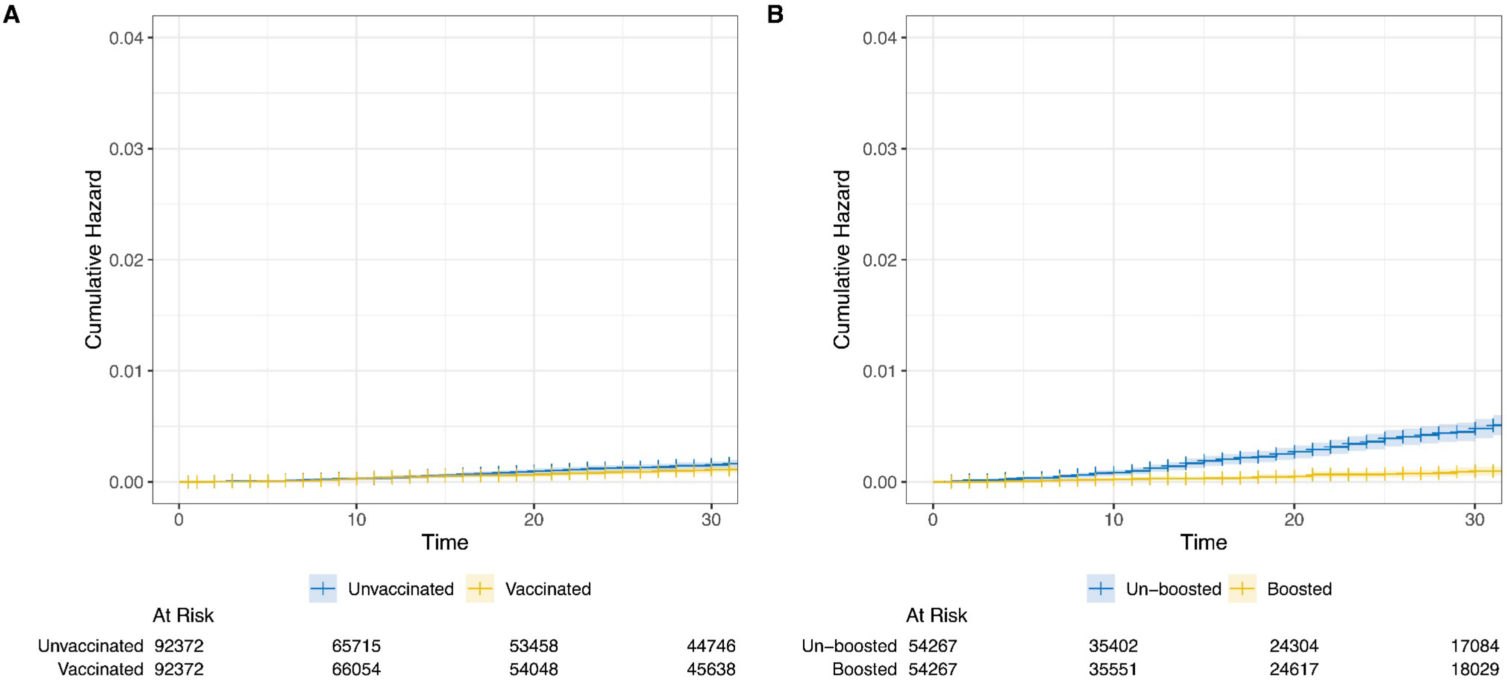

SUPPL. FIGURE 6: Forest plot of estimated COVID-19 vaccine effectiveness (point estimate) and its 95% confidence interval (error-bars) among subgroups for the primary vaccination (Cohort A), including number of events, observations, and 95% vaccine effectiveness confidence interval. Counts below five have been masked to protect patients privacy. VE = Vaccine Effectiveness.

	Vaccination Status	Events/Obs Unvax	Events/Obs Vax	VE (95%CI)	Interaction p–value
Age					
< 65 years	Partially Vaccinated	180 / 42103	20 / 30515	64.4% (37.4 – 79.8)	0.000
< 65 years	Fully Vaccinated	180 / 42103	29 / 20454	74.7% (59.2 – 84.3)	
>= 65 years	Partially Vaccinated	185 / 50269	39 / 28588	10.6% (–28.0 – 42.4)	
>= 65 years	Fully Vaccinated	185 / 50269	90 / 17857	33.2% (8.8 – 51.0)	
Sex					
Male	Partially Vaccinated	200 / 45355	34 / 27692	39.5% (4.8 – 61.6)	0.337
Male	Fully Vaccinated	200 / 45355	77 / 17371	43.6% (22.1 – 59.2)	
Female	Partially Vaccinated	165 / 47017	25 / 31411	45.4% (8.1 – 67.6)	
Female	Fully Vaccinated	165 / 47017	42 / 20940	62.0% (42.6 – 74.9)	
Cancer Diagnosis Time					
< 1 year	Partially Vaccinated	129 / 23985	22 / 16667	42.3% (–0.7 – 67.0)	0.197
< 1 year	Fully Vaccinated	129 / 23985	31 / 10877	65.1% (43.8 – 78.3)	
1 – 5 years	Partially Vaccinated	236 / 68387	37 / 42436	42.1% (10.7 – 62.5)	
1 – 5 years	Fully Vaccinated	236 / 68387	88 / 27434	44.7% (25.3 – 59.1)	
Metastatic Disease					
No	Partially Vaccinated	273 / 78520	47 / 49775	39.2% (10.1 – 58.9)	0.025
No	Fully Vaccinated	273 / 78520	76 / 32271	59.6% (44.7 – 70.4)	
Yes	Partially Vaccinated	92 / 13852	12 / 9328	48.1% (–7.8 – 75.2)	
Yes	Fully Vaccinated	92 / 13852	43 / 6040	24.0% (–15.4 – 51.1)	
Lung Cancer					
No	Partially Vaccinated	326 / 86881	56 / 55700	38.9% (12.3 – 57.5)	0.642
No	Fully Vaccinated	326 / 86881	107 / 36166	52.0% (37.0 – 63.4)	
Yes	Partially Vaccinated	39 / 5491	< 5 / 3403	68.3% (–23.8 – 92.3)	
Yes	Fully Vaccinated	39 / 5491	12 / 2145	45.4% (–16.1 – 75.0)	
Hemathological Cancer					
No	Partially Vaccinated	296 / 86355	50 / 54513	39.8% (12.0 – 58.8)	0.646
No	Fully Vaccinated	296 / 86355	102 / 35198	49.1% (32.7 – 61.5)	
Yes	Partially Vaccinated	69 / 6017	9 / 4590	47.1% (–19.2 – 77.4)	
Yes	Fully Vaccinated	69 / 6017	17 / 3113	62.7% (29.0 – 80.3)	
Variant of Concern					
Other	Partially Vaccinated	355 / 92372	59 / 59103	42.3% (17.9 – 59.4)	0.145
Other	Fully Vaccinated	355 / 92372	107 / 38241	54.3% (40.2 – 65.1)	
Delta	Partially Vaccinated	10 / 14353	0 / 489	100.0% (–100.0 – 100.0)	
Delta	Fully Vaccinated	10 / 14353	12 / 15915	–4.2% (–63.0 – 59.7)	

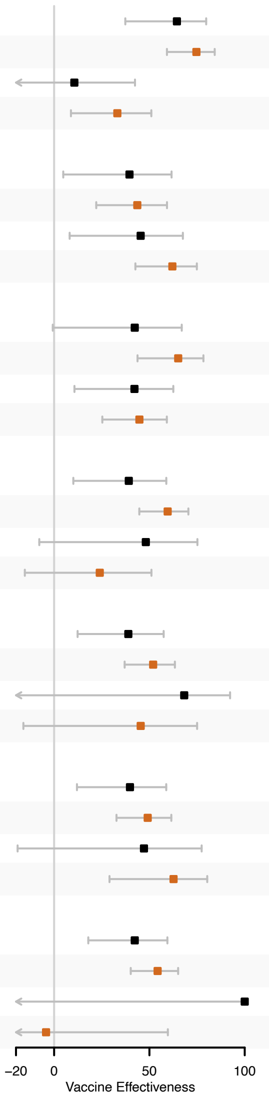

Black = Partially Vaccinated. Orange = Fully Vaccinated.

SUPPL. FIGURE 7: . Forest plot of estimated COVID-19 vaccine effectiveness (point estimate) and its 95% confidence interval (error-bars) among subgroups for booster vaccination (Cohort A), including number of events, observations, and 95% vaccine effectiveness confidence interval. Counts below five have been masked to protect patients privacy. rVE = Relative Vaccine Effectiveness.
Black = Booster 14 – 60 days. Orange = Booster 60 days or more

	Vaccination Status	Events/Obs Unboosted	Events/Obs Boosted	rVE (95%CI)	Interaction p–value
Age					
< 65 years	Booster 14 – 60 days	47 / 18167	13 / 9817	49.3% (−6.9 – 76.1)	0.028
< 65 years	Booster 60 days+	47 / 18167	14 / 5450	40.9% (−19.0 – 71.7)	
>= 65 years	Booster 14 – 60 days	313 / 36100	30 / 19052	82.2% (71.6 – 88.9)	
>= 65 years	Booster 60 days+	313 / 36100	74 / 6339	46.9% (24.3 – 62.7)	
Sex					
Male	Booster 14 – 60 days	256 / 27614	26 / 14168	81.4% (69.2 – 88.7)	0.006
Male	Booster 60 days+	256 / 27614	48 / 5356	58.6% (37.5 – 72.6)	
Female	Booster 14 – 60 days	104 / 26653	17 / 14701	69.8% (43.2 – 83.9)	
Female	Booster 60 days+	104 / 26653	40 / 6433	16.7% (−25.1 – 48.0)	
Cancer Diagnosis Time					
< 1 year	Booster 14 – 60 days	99 / 11604	9 / 6576	83.7% (62.6 – 92.9)	0.777
< 1 year	Booster 60 days+	99 / 11604	24 / 2987	47.6% (7.8 – 70.2)	
1 – 5 years	Booster 14 – 60 days	261 / 42663	34 / 22293	75.7% (61.9 – 84.5)	
1 – 5 years	Booster 60 days+	261 / 42663	64 / 8802	45.2% (20.4 – 62.3)	
Metastatic Disease					
No	Booster 14 – 60 days	269 / 47868	33 / 25487	77.7% (64.9 – 85.8)	0.972
No	Booster 60 days+	269 / 47868	68 / 10406	45.2% (21.0 – 62.0)	
Yes	Booster 14 – 60 days	91 / 6399	10 / 3382	76.9% (48.8 – 89.6)	
Yes	Booster 60 days+	91 / 6399	20 / 1383	42.2% (−5.8 – 68.5)	
Lung Cancer					
No	Booster 14 – 60 days	305 / 51550	41 / 27497	75.2% (62.5 – 83.6)	0.151
No	Booster 60 days+	305 / 51550	80 / 11272	42.6% (19.0 – 59.3)	
Yes	Booster 14 – 60 days	55 / 2717	< 5 / 1372	92.9% (61.1 – 98.7)	
Yes	Booster 60 days+	55 / 2717	8 / 517	64.6% (11.9 – 85.8)	
Hemathological Cancer					
No	Booster 14 – 60 days	307 / 50957	36 / 27144	78.5% (66.8 – 86.1)	0.783
No	Booster 60 days+	307 / 50957	74 / 11101	47.0% (24.6 – 62.8)	
Yes	Booster 14 – 60 days	53 / 3310	7 / 1725	72.8% (29.3 – 89.6)	
Yes	Booster 60 days+	53 / 3310	14 / 688	31.7% (−29.5 – 67.1)	
Variant of Concern					
Delta	Booster 14 – 60 days	301 / 54267	43 / 28869	77.9% (67.0 – 85.2)	0.026
Delta	Booster 60 days+	301 / 54267	29 / 11789	62.8% (36.6 – 78.1)	
Omicron	Booster 14 – 60 days	59 / 7871	NA / NA	NA% (NA – NA)	
Omicron	Booster 60 days+	59 / 7871	59 / 10418	29.3% (−1.4 – 50.7)	
Previous mRNA Vaccine					
No	Booster 14 – 60 days	65 / 12814	7 / 5482	80.5% (49.7 – 92.4)	0.155
No	Booster 60 days+	65 / 12814	8 / 2299	73.7% (35.0 – 89.3)	
Yes	Booster 14 – 60 days	295 / 41453	36 / 23387	77.4% (65.0 – 85.3)	
Yes	Booster 60 days+	295 / 41453	80 / 9490	39.4% (14.4 – 57.1)	

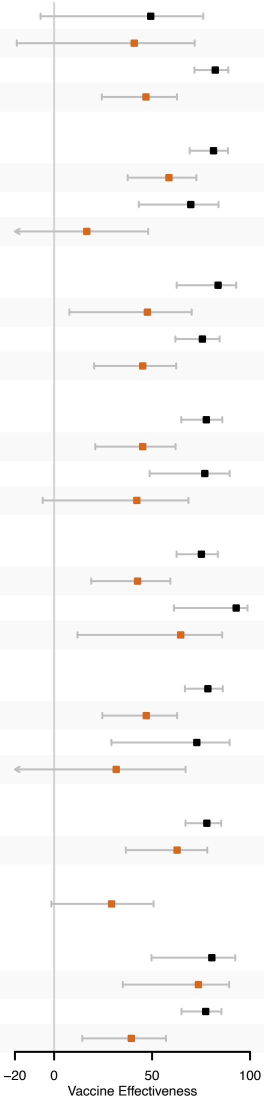

SUPPL. FIGURE 8. Forest plot of the estimated hazard ratio (HR) and its 95% confidence interval of vaccinated versus unvaccinated (reference group) for negative control outcomes by status and time after vaccination (Figure A). The size of the point represents the number of events recorded. Outcomes with zero events were excluded from the plot. Figure B shows the HR and its 95% confidence interval as precise numbers.

A

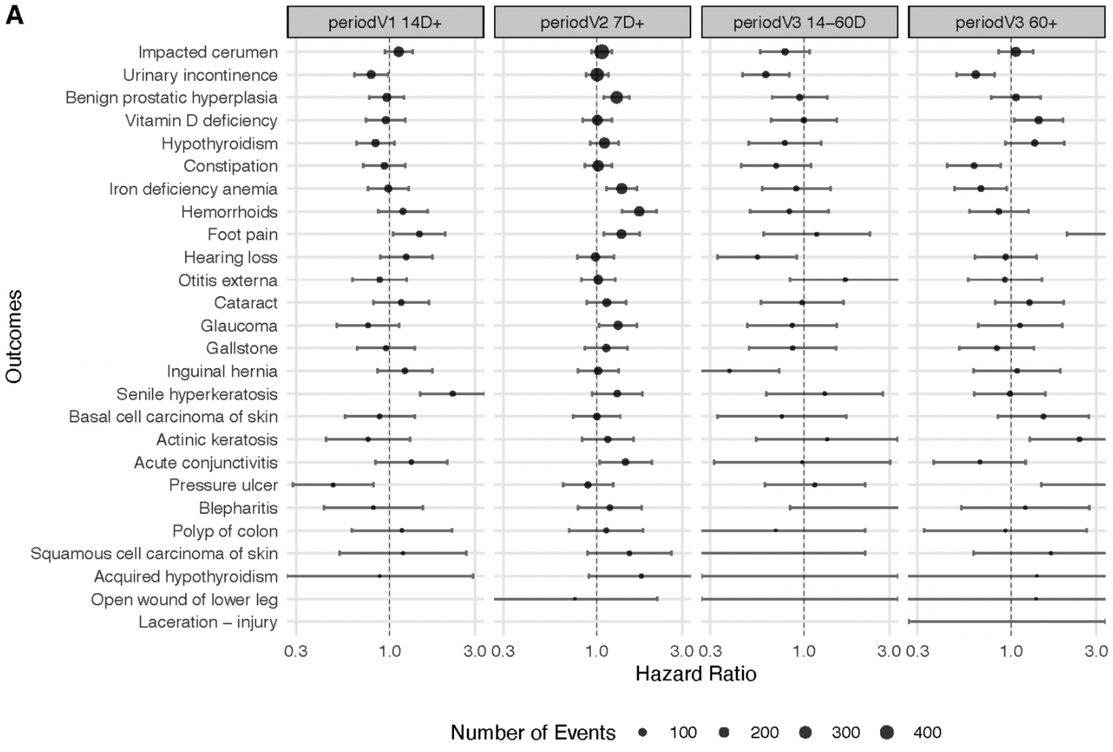

B

Outcomes	periodV1 14D+	periodV2 7D+	periodV3 14–60D	periodV3 60+
Impacted cerumen	1.12 (0.94 – 1.34)	1.07 (0.94 – 1.21)	0.78 (0.57 – 1.08)	1.06 (0.85 – 1.33)
Urinary incontinence	0.79 (0.63 – 0.98)	1.01 (0.87 – 1.16)	0.61 (0.45 – 0.83)	0.64 (0.50 – 0.81)
Benign prostatic hyperplasia	0.96 (0.77 – 1.21)	1.29 (1.10 – 1.52)	0.95 (0.66 – 1.35)	1.06 (0.77 – 1.46)
Vitamin D deficiency	0.95 (0.74 – 1.23)	1.01 (0.83 – 1.22)	1.00 (0.66 – 1.52)	1.43 (1.05 – 1.95)
Hypothyroidism	0.83 (0.65 – 1.07)	1.10 (0.92 – 1.32)	0.78 (0.49 – 1.25)	1.36 (0.93 – 1.98)
Constipation	0.93 (0.71 – 1.22)	1.02 (0.85 – 1.21)	0.70 (0.45 – 1.10)	0.62 (0.44 – 0.88)
Iron deficiency anemia	0.98 (0.75 – 1.28)	1.38 (1.14 – 1.67)	0.91 (0.58 – 1.41)	0.68 (0.48 – 0.95)
Hemorrhoids	1.19 (0.86 – 1.63)	1.73 (1.38 – 2.16)	0.83 (0.50 – 1.37)	0.86 (0.59 – 1.25)
Foot pain	1.46 (1.05 – 2.05)	1.37 (1.09 – 1.73)	1.18 (0.59 – 2.34)	3.57 (2.06 – 6.19)
Hearing loss	1.24 (0.88 – 1.74)	0.98 (0.78 – 1.24)	0.55 (0.33 – 0.92)	0.93 (0.63 – 1.39)
Otitis externa	0.88 (0.62 – 1.25)	1.02 (0.82 – 1.27)	1.71 (0.84 – 3.47)	0.92 (0.57 – 1.48)
Cataract	1.16 (0.81 – 1.65)	1.14 (0.88 – 1.46)	0.98 (0.57 – 1.67)	1.27 (0.82 – 1.97)
Glaucoma	0.76 (0.51 – 1.13)	1.31 (1.03 – 1.68)	0.86 (0.48 – 1.52)	1.12 (0.65 – 1.93)
Gallstone	0.95 (0.66 – 1.38)	1.13 (0.86 – 1.48)	0.86 (0.49 – 1.52)	0.83 (0.52 – 1.35)
Inguinal hernia	1.22 (0.85 – 1.74)	1.02 (0.78 – 1.32)	0.38 (0.20 – 0.73)	1.08 (0.62 – 1.89)
Senile hyperkeratosis	2.25 (1.48 – 3.41)	1.30 (0.94 – 1.79)	1.31 (0.62 – 2.76)	0.99 (0.62 – 1.56)
Basal cell carcinoma of skin	0.88 (0.56 – 1.38)	1.00 (0.74 – 1.35)	0.75 (0.33 – 1.72)	1.52 (0.85 – 2.73)
Actinic keratosis	0.76 (0.44 – 1.29)	1.15 (0.83 – 1.60)	1.35 (0.54 – 3.35)	2.41 (1.27 – 4.57)
Acute conjunctivitis	1.32 (0.83 – 2.10)	1.45 (1.03 – 2.02)	0.98 (0.31 – 3.03)	0.67 (0.37 – 1.21)
Pressure ulcer	0.48 (0.29 – 0.81)	0.89 (0.65 – 1.23)	1.15 (0.60 – 2.20)	3.90 (1.47 – 10.35)
Blepharitis	0.81 (0.43 – 1.53)	1.18 (0.79 – 1.78)	3.94 (0.84 – 18.56)	1.20 (0.53 – 2.74)
Polyp of colon	1.17 (0.62 – 2.23)	1.13 (0.70 – 1.82)	0.70 (0.22 – 2.19)	0.93 (0.33 – 2.65)
Squamous cell carcinoma of skin	1.19 (0.53 – 2.68)	1.52 (0.88 – 2.62)	0.25 (0.03 – 2.21)	1.67 (0.62 – 4.52)
Acquired hypothyroidism	0.88 (0.26 – 2.92)	1.77 (0.91 – 3.48)		1.40 (0.23 – 8.35)
Open wound of lower leg		0.76 (0.26 – 2.18)		1.38 (0.23 – 8.25)

Hazard Ratio (95% Confidence Interval)

SUPPL. FIGURE 9. Funnel plot including estimated hazard ratios (HR) for negative control outcomes by timing and status of vaccination: partially vaccinated (A), fully vaccinated (B), 14 to 60 days after booster (C), and 60 days or more after booster dose (D).

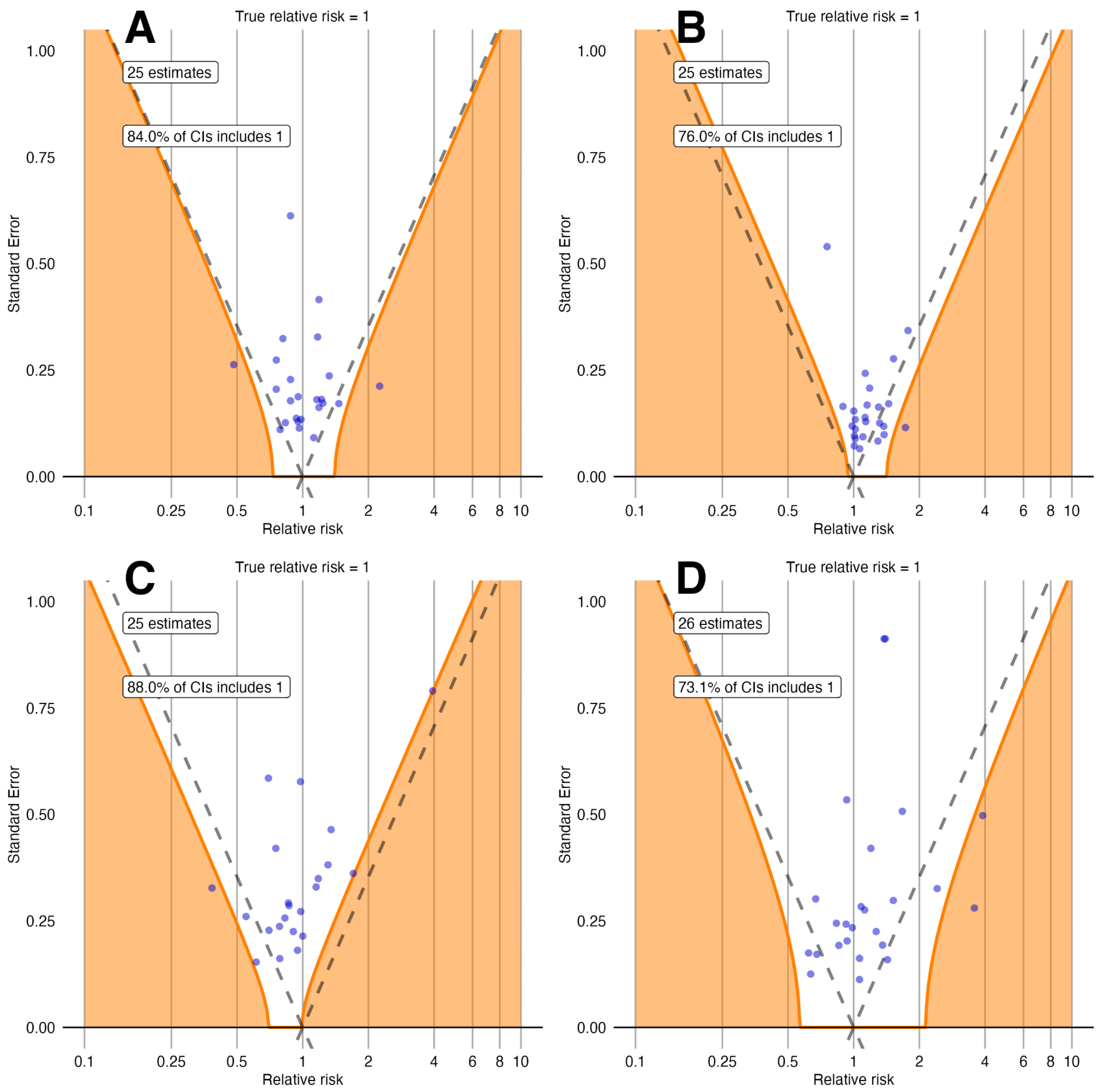

SUPPL. FIGURE 10. Cumulative hazards curves of all-cause hospitalizations (Figure A), non-COVID-19 death (Figure B), and COVID-19 hospitalization (Figure C) outcomes for the original (left) and the restricted matching cohort (right) for the primary vaccination cohort (Cohort A). The solid lines represent the estimated cumulative hazards, while the shaded areas indicate the 95% confidence intervals.

A

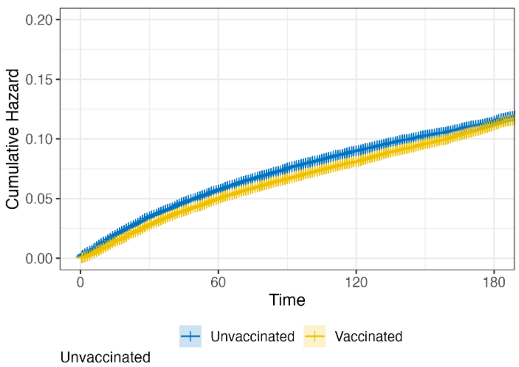

**Unvaccinated**				
At Risk	92372	30860	21974	13257
Interval Events	0	2972	857	444
**Vaccinated**				
At Risk	92372	32350	23805	14771
Interval Events	0	2507	861	596

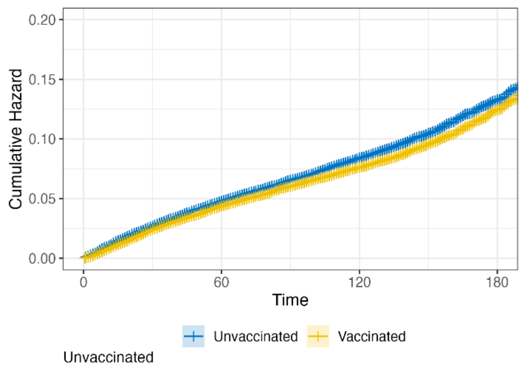

**Unvaccinated**				
At Risk	84308	25212	17382	9669
Interval Events	0	2070	754	649
**Vaccinated**				
At Risk	84308	26185	18680	10804
Interval Events	0	1849	713	696

B

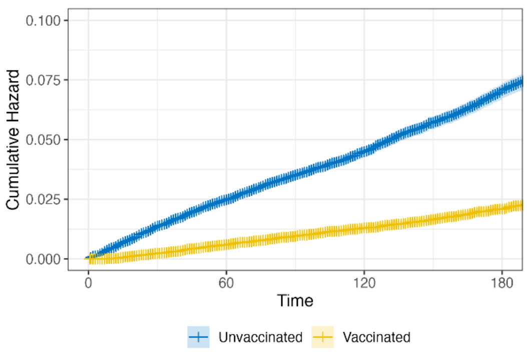

**Unvaccinated**				
At Risk	92372	32134	22811	13868
Interval Events	0	1249	544	473
**Vaccinated**				
At Risk	92372	33514	24884	15794
Interval Events	0	266	202	161

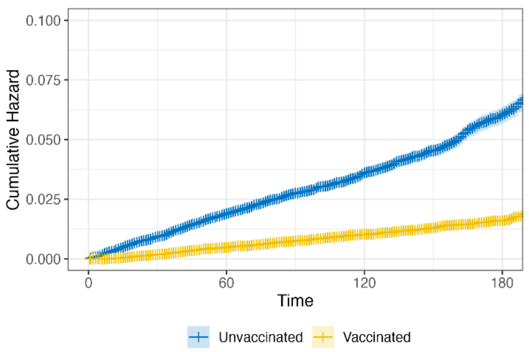

**Unvaccinated**				
At Risk	84308	25955	17782	10031
Interval Events	0	799	362	332
**Vaccinated**				
At Risk	84308	26927	19341	11445
Interval Events	0	179	125	89

C

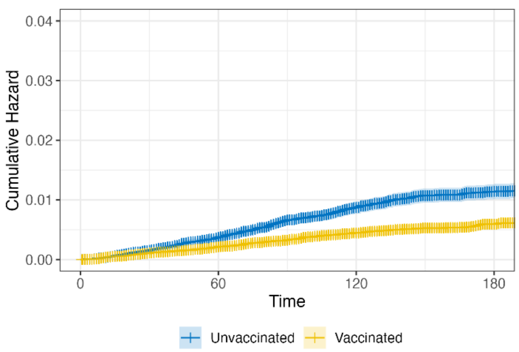

**Unvaccinated**				
At Risk	92372	31985	22674	13804
Interval Events	0	169	135	53
**Vaccinated**				
At Risk	92372	33442	24791	15747
Interval Events	0	104	67	30

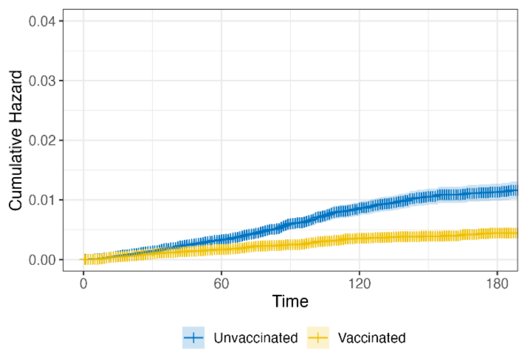

**Unvaccinated**				
At Risk	84308	25814	17672	9978
Interval Events	0	129	112	40
**Vaccinated**				
At Risk	84308	26871	19282	11414
Interval Events	0	72	42	13

SUPPL. FIGURE 11. Cumulative hazards curves of all-cause hospitalizations (Figure A), non-COVID-19 death (Figure B), and COVID-19 hospitalization (Figure C) outcomes for the original (left) and the restricted matching cohort (right) for the booster vaccination cohort (Cohort B). The solid lines represent the estimated cumulative hazards while the shaded areas indicate the 95% confidence intervals.

A

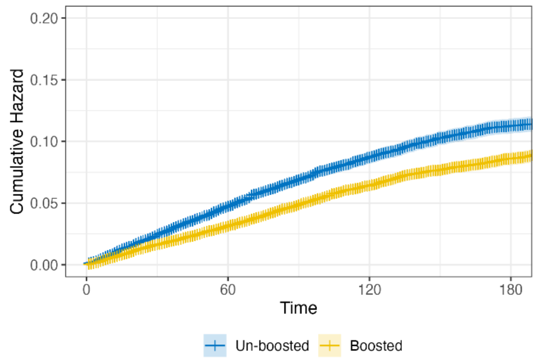

Un-boosted				
At Risk	54267	11311	9474	5148
Interval Events	0	1057	407	215
Boosted				
At Risk	54267	12208	10544	6106
Interval Events	0	715	367	206

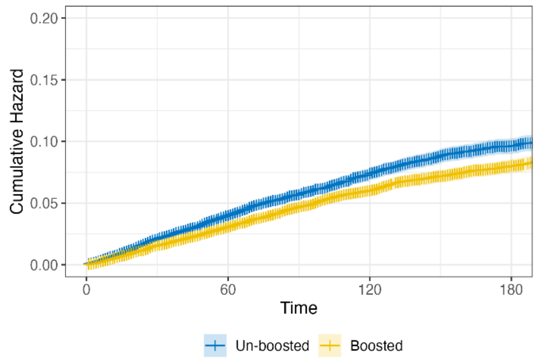

Un-boosted				
At Risk	46930	8911	7560	3911
Interval Events	0	719	276	150
Boosted				
At Risk	46930	9424	8188	4466
Interval Events	0	543	257	146

B

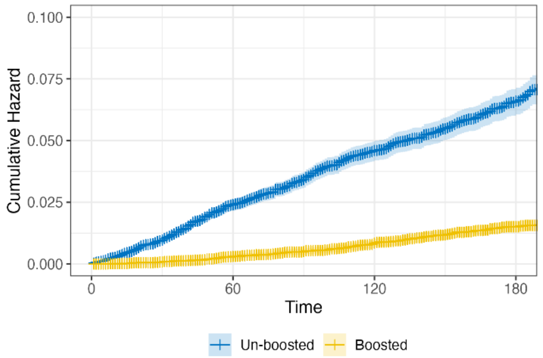

Un-boosted				
At Risk	54267	9894	7394	3682
Interval Events	0	479	182	120
Boosted				
At Risk	54267	11954	9990	5671
Interval Events	0	52	57	58

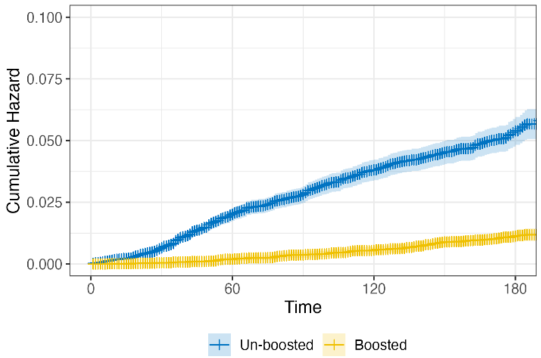

Un-boosted				
At Risk	46930	7658	5770	2697
Interval Events	0	286	116	71
Boosted				
At Risk	46930	9196	7744	4168
Interval Events	0	25	30	38

C

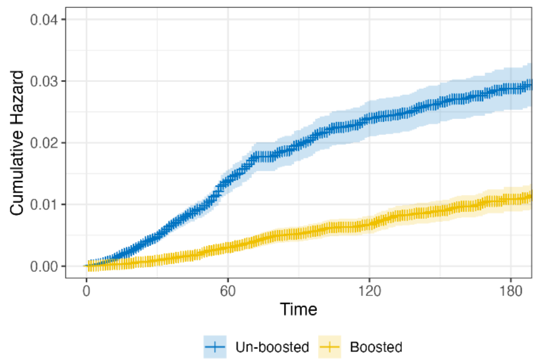

Un-boosted				
At Risk	54267	9620	7324	3651
Interval Events	0	240	85	29
Boosted				
At Risk	54267	11859	9941	5617
Interval Events	0	55	40	36

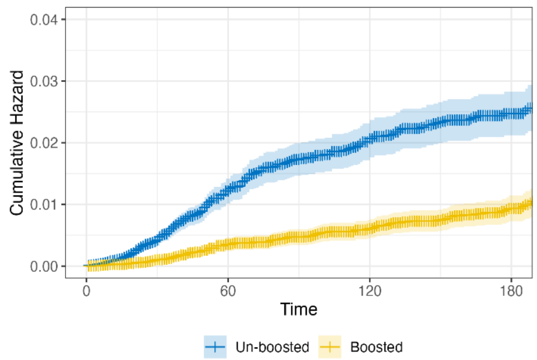

Un-boosted				
At Risk	46930	7436	5709	2677
Interval Events	0	170	55	20
Boosted				
At Risk	46930	9110	7714	4137
Interval Events	0	49	21	21

SUPPL. FIGURE 12. Forest plot of COVID-19 vaccine effectiveness (point estimate) and its 95% confidence interval for the main results and respective sensitivity analysis for primary vaccination (Cohort A)outcome of COVID-19 hospitalization. Sensitivity analysis included: (i) only patients with any COVID-19 test from 27th December 2020 to 30th June 2022)(tested patients); (ii) additionally excluded patients with undefined skin cancer (strict cancer diagnosis); (iii) only PCR-confirmed diagnosis (laboratory COVID-19 diagnosis); (iv) COVID-19 from 21 days before up to 3 days after hospital admission, (v) COVID-19 from 14 days before up to 3 days after hospital admission; and (vi) a sensitivity analysis excluding Ad26.COV2.S vaccine product.

PRIMARY VACCINATION

**Main Results**	**Person–Days**	**No Events**	**VE**
Unvaccinated	6,130,100	365	Reference
Partially Vaccinated	1,497,372	59	42.0% (22.3 – 56.7)
Fully Vaccinated	3,972,188	119	51.8% (40.3 – 61.1)
**Only Tested Patients**			
Unvaccinated	6,762,172	388	Reference
Partially Vaccinated	1,416,239	59	40.6% (20.3 – 55.7)
Fully Vaccinated	3,735,212	115	51.4% (39.5 – 60.9)
**Strict Cancer Diagnosis**			
Unvaccinated	5,021,660	378	Reference
Partially Vaccinated	1,197,080	59	46.8% (28.7 – 60.4)
Fully Vaccinated	3,122,830	118	56.5% (46.0 – 65.0)
**Laboratory COVID–19**			
Unvaccinated	7,180,253	394	Reference
Partially Vaccinated	1,498,013	59	40.7% (20.5 – 55.8)
Fully Vaccinated	3,980,425	119	50.8% (39.1 – 60.3)
**COVID–19 21D bef. – 3D aft.**			
Unvaccinated	7,160,925	390	Reference
Partially Vaccinated	1,497,475	57	43.2% (23.4 – 57.8)
Fully Vaccinated	3,972,353	122	49.4% (37.3 – 59.1)
**COVID–19 14D bef. – 3D aft.**			
Unvaccinated	7,161,126	383	Reference
Partially Vaccinated	1,497,490	56	43.3% (23.4 – 58.0)
Fully Vaccinated	3,972,481	117	50.2% (38.1 – 59.9)
**Excl. Ad26.COV2.S Vaccine**			
Unvaccinated	6,842,442	387	Reference
Partially Vaccinated	1,247,284	51	39.9% (17.3 – 56.3)
Fully Vaccinated	3,971,402	119	53.3% (42.1 – 62.3)

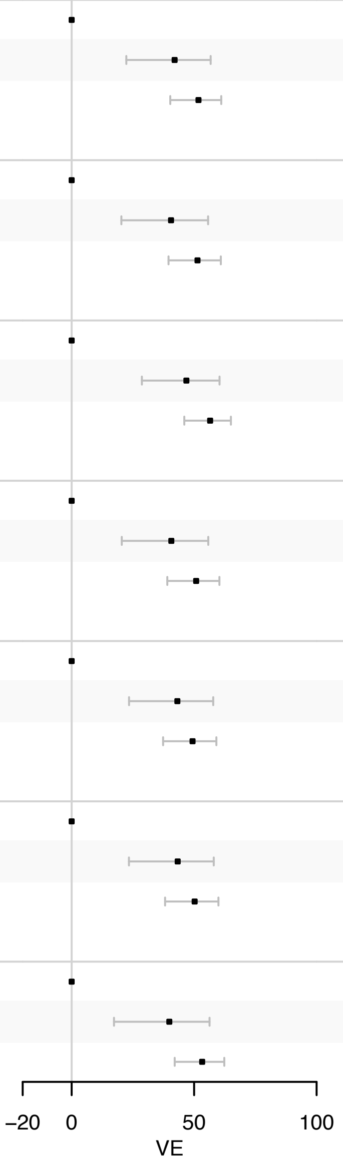

SUPPL. FIGURE 13. Forest plot of COVID-19 vaccine effectiveness (point estimate) and its 95% confidence interval for the main results and respective sensitivity analysis for booster vaccination (Cohort B) of primary outcome of COVID-19 hospitalization. Sensitivity analysis included: (i) only patients with any COVID-19 test from 27th December 2020 to 30th June 2022)(tested patients); (ii) additionally excluded patients with undefined skin cancer (strict cancer diagnosis); (iii) only PCR-confirmed diagnosis (laboratory COVID-19 diagnosis); (iv) COVID-19 from 21 days before up to 3 days after hospital admission, (v) COVID-19 from 14 days before up to 3 days after hospital admission.

BOOSTER VACCINATION

Main Results	Person−Days	No Events	rVE
Un−boosted	2,230,862	360	Reference
14 – 59 days after booster	786,371	43	77.9% (69.2 – 84.2)
60 or more days after booster	1,281,426	88	45.8% (28.7 – 58.9)
**Only Tested Patients**			
Un−boosted	2,621,218	346	Reference
14 – 59 days after booster	734,846	41	78.0% (69.1 – 84.4)
60 or more days after booster	1,199,910	83	42.5% (23.3 – 56.8)
**Strict Cancer Diagnosis**			
Un−boosted	2,063,754	357	Reference
14 – 59 days after booster	597,575	43	77.1% (67.9 – 83.6)
60 or more days after booster	944,915	88	47.3% (30.6 – 60.1)
**Laboratory COVID−19**			
Un−boosted	2,852,410	369	Reference
14 – 59 days after booster	786,832	43	77.6% (68.7 – 83.9)
60 or more days after booster	1,302,088	87	44.0% (26.1 – 57.5)
**COVID−19 21D bef. – 3D aft.**			
Un−boosted	2,804,241	359	Reference
14 – 59 days after booster	786,370	43	77.5% (68.5 – 83.9)
60 or more days after booster	1,281,492	87	42.7% (24.2 – 56.7)
**COVID−19 14D bef. – 3D aft.**			
Un−boosted	2,804,355	353	Reference
14 – 59 days after booster	786,343	41	78.0% (69.1 – 84.4)
60 or more days after booster	1,281,505	84	43.7% (25.2 – 57.6)

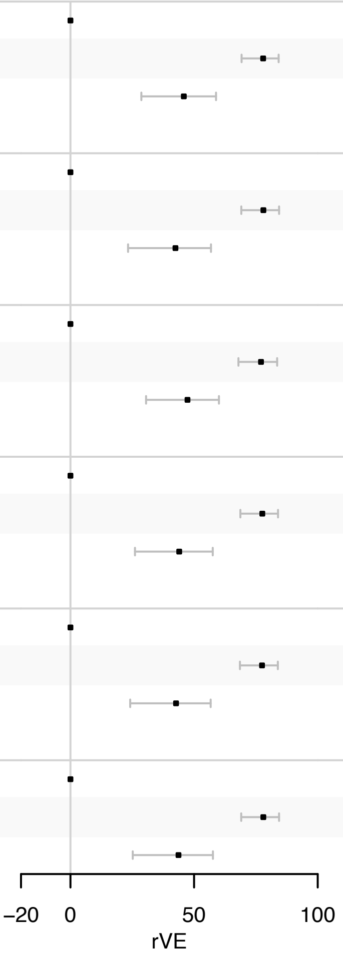

SUPPL. FIGURE 14. Rolling Entry Matching Scheme and Target-Trial Emulation Framework (Illustration)

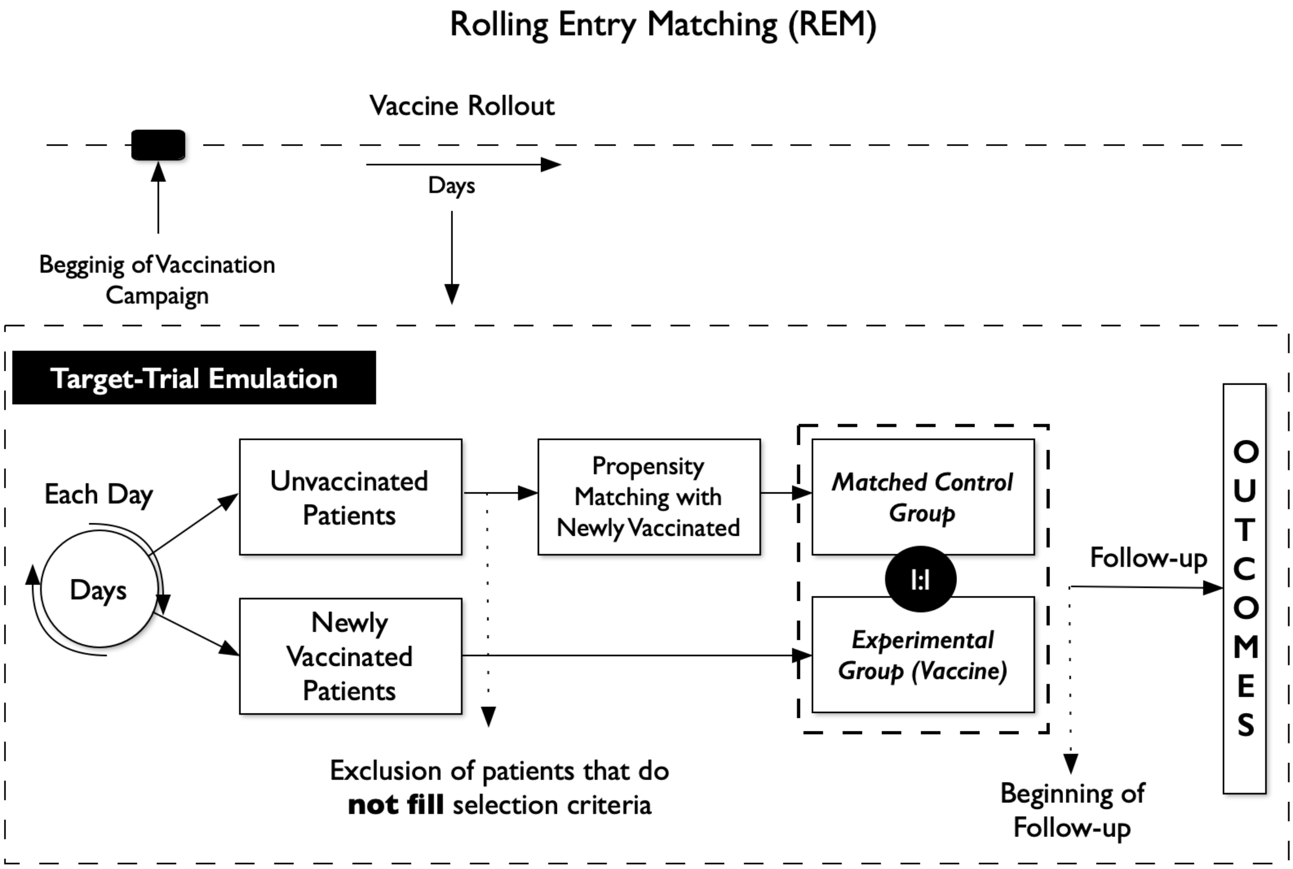

SUPPL. TABLE 1. Baseline characteristics of matched and un-matched patients for the primary vaccination cohort (cohort A).

	Un-matched	Matched
**Number of Patients**	34047	92372
**Age, Mean (SD)**	62.93 (18.55)	64.58 (15.18)
**Age Group, N (%)**		
18-49	8244 (24.2)	15948 (17.3)
50-59	5494 (16.1)	15982 (17.3)
60-69	6757 (19.8)	23078 (25.0)
70-79	6920 (20.3)	23350 (25.3)
80-115	6632 (19.5)	14014 (15.2)
**Female Sex, N (%)**	17647 (51.8)	47017 (50.9)
**MEDEA deprivation index, N (%)**		
Missing	6328 (18.6)	10459 (11.3)
Rural	5394 (15.8)	13765 (14.9)
Urban, Quintile 1	4601 (13.5)	15154 (16.4)
Urban, Quintile 2	4426 (13.0)	14353 (15.5)
Urban, Quintile 3	4601 (13.5)	13624 (14.7)
Urban, Quintile 4	4440 (13.0)	13448 (14.6)
Urban, Quintile 5	4257 (12.5)	11569 (12.5)
**Time Since Diagnosis (Years), N (%)**		
Zero	10897 (32.0)	29731 (32.2)
One	5322 (15.6)	19126 (20.7)
Two	4550 (13.4)	16990 (18.4)
Three	4282 (12.6)	16113 (17.4)
Four	8996 (26.4)	10412 (11.3)
**Cancer Diagnosis, N (%)**		
Breast	5153 (15.1)	15690 (17.0)
Prostate	3196 (9.4)	11338 (12.3)
Colorectal	4252 (12.5)	12125 (13.1)
Lung	2458 (7.2)	5198 (5.6)
Head and Neck	1023 (3.0)	2703 (2.9)
Endometrium	630 (1.9)	1929 (2.1)
Cervix/Uterus	864 (2.5)	1835 (2.0)
Bladder	2698 (7.9)	8209 (8.9)
Biliary/CHC	851 (2.5)	1653 (1.8)
Melanoma	1198 (3.5)	3597 (3.9)
Pancreas	749 (2.2)	1342 (1.5)
Kidney	1081 (3.2)	3382 (3.7)
Gastric	709 (2.1)	1502 (1.6)
Esophagus	230 (0.7)	477 (0.5)
Testis	357 (1.0)	607 (0.7)
Thyroid	760 (2.2)	1667 (1.8)
CNS	526 (1.5)	912 (1.0)
Neuroendocrine Cancer (NEC)	164 (0.5)	429 (0.5)
Sarcomas	447 (1.3)	920 (1.0)

Leukemia	998 (2.9)	2431 (2.6)
Myeloma	355 (1.0)	1131 (1.2)
Lymphoma	1215 (3.6)	3170 (3.4)
Hematological	2507 (7.4)	6611 (7.2)
Other	3227 (9.5)	7889 (8.5)
**Metastatic Solid Tumor, N (%)**	6757 (19.8)	13507 (14.6)
**Charlson Comorbidity Index, Median (IQR)**	3.00 [2.00, 7.00]	3.00 [2.00, 6.00]
**Comorbidities, N (%)**		
Reumatological Disease	971 (2.9)	2736 (3.0)
Malignancy	34047 (100.0)	92372 (100.0)
Mild Diabetes	7093 (20.8)	20742 (22.5)
Mild Liver Disease	1217 (3.6)	2921 (3.2)
Peptic Ulcer Disease	1874 (5.5)	5342 (5.8)
Chronic Pulmonary Disease	5988 (17.6)	16438 (17.8)
Chronic Renal Disease	6489 (19.1)	15767 (17.1)
Cerebrovascular Disease	2321 (6.8)	5976 (6.5)
Diabetes with Complications	2243 (6.6)	5852 (6.3)
Congestive Heart Failure (CHF)	3272 (9.6)	7284 (7.9)
Dementia	1099 (3.2)	2342 (2.5)
Peripheral Artery Disease	1823 (5.4)	4885 (5.3)
Myocardial Infarction	1484 (4.4)	4124 (4.5)
Moderate/Severe Liver Disease	827 (2.4)	1555 (1.7)
Hemiplegia/Paraplegia	663 (1.9)	1344 (1.5)
AIDS/HIV	281 (0.8)	517 (0.6)
**Number of Outpatient Visits*, N (%)**		
0	5697 (16.7)	9029 (9.8)
1	3330 (9.8)	8998 (9.7)
2	3139 (9.2)	8694 (9.4)
3+	21881 (64.3)	65651 (71.1)

* In the previous year - 28th December 2019 until 27th December 2020

SUPPL. TABLE 2. Baseline characteristics of matched and un-matched patients for the booster vaccination cohort (cohort B).

	Un-matched	Matched
**Number of Patients**	22291	54267
**Age, Mean (SD)**	59.66 (18.21)	69.63 (12.43)
**Age Group, N (%)**		
18-49	6781 (30.4)	3677 (6.8)
50-59	4509 (20.2)	8184 (15.1)
60-69	4401 (19.7)	14174 (26.1)
70-79	3276 (14.7)	16575 (30.5)
80-115	3324 (14.9)	11657 (21.5)
**Female Sex, N(%)**	12089 (54.2)	26653 (49.1)
**MEDEA (2001), N (%)**		
Missing	3010 (13.5)	4972 (9.2)
Rural	4149 (18.6)	8430 (15.5)
Urban, Quintile 1	3096 (13.9)	9469 (17.4)
Urban, Quintile 2	3077 (13.8)	8841 (16.3)
Urban, Quintile 3	3077 (13.8)	8164 (15.0)
Urban, Quintile 4	3110 (14.0)	7946 (14.6)
Urban, Quintile 5	2772 (12.4)	6445 (11.9)
**Time Since Diagnosis (Years), N (%)**		
Zero	6149 (27.6)	11604 (21.4)
One	4409 (19.8)	11263 (20.8)
Two	4167 (18.7)	10869 (20.0)
Three	3873 (17.4)	10254 (18.9)
Four	3693 (16.6)	10277 (18.9)
**Cancer Diagnosis, N (%)**		
Breast	3959 (17.8)	9137 (16.8)
Prostate	1948 (8.7)	8036 (14.8)
Colorectal	2550 (11.4)	7706 (14.2)
Lung	1190 (5.3)	2509 (4.6)
Head and Neck	644 (2.9)	1539 (2.8)
Endometrium	478 (2.1)	1182 (2.2)
Cervix/Uterus	515 (2.3)	971 (1.8)
Bladder	1674 (7.5)	5453 (10.0)
Biliary/CHC	395 (1.8)	791 (1.5)
Melanoma	913 (4.1)	2219 (4.1)
Pancreas	318 (1.4)	576 (1.1)
Kidney	730 (3.3)	2064 (3.8)
Gastric	334 (1.5)	817 (1.5)
Esophagus	120 (0.5)	224 (0.4)
Testis	298 (1.3)	186 (0.3)
Thyroid	541 (2.4)	828 (1.5)
CNS	321 (1.4)	301 (0.6)
Neuroendocrine Cancer (NEC)	108 (0.5)	230 (0.4)
Sarcomas	259 (1.2)	461 (0.8)

Leukemia	594 (2.7)	1259 (2.3)
Myeloma	292 (1.3)	462 (0.9)
Lymphoma	888 (4.0)	1437 (2.6)
Hematological	1760 (7.9)	3106 (5.7)
Other	2344 (10.5)	4122 (7.6)
**Metastatic Solid Tumor, N (%)**	3449 (15.5)	5952 (11.0)
**Charlson Comorbidity Index, Median (IQR)**	3.00 [2.00, 5.00]	3.00 [2.00, 5.00]
**Comorbidities, N (%)**		
Reumatological Disease	543 (2.4)	1768 (3.3)
Malignancy	22291 (100.0)	54267 (100.0)
Mild Diabetes	4038 (18.1)	13295 (24.5)
Mild Liver Disease	662 (3.0)	1488 (2.7)
Peptic Ulcer Disease	1002 (4.5)	3268 (6.0)
Chronic Pulmonary Disease	3289 (14.8)	10137 (18.7)
Chronic Renal Disease	3305 (14.8)	9997 (18.4)
Cerebrovascular Disease	1202 (5.4)	3889 (7.2)
Diabetes with Complications	1181 (5.3)	3611 (6.7)
Congestive Heart Failure (CHF)	1558 (7.0)	4566 (8.4)
Dementia	475 (2.1)	1572 (2.9)
Peripheral Artery Disease	928 (4.2)	3098 (5.7)
Myocardial Infarction	830 (3.7)	2673 (4.9)
Moderate/Severe Liver Disease	365 (1.6)	653 (1.2)
Hemiplegia/Paraplegia	303 (1.4)	735 (1.4)
AIDS/HIV	161 (0.7)	199 (0.4)
**Number of Outpatient Visits*, N (%)**		
0	2933 (13.2)	4380 (8.1)
1	2356 (10.6)	5137 (9.5)
2	2225 (10.0)	5025 (9.3)
3+	14777 (66.3)	39725 (73.2)

* In the previous year - 28th December 2019 until 27th December 2020

SUPPL. TABLE 3. COVID-19 Hospitalization Outcome Vaccine Effectiveness from the cause-specific (main results) and competing risk (subdistribution HR, Fine-Gray model) models

	VE (from HR) [cause-specific model]	VE (from subdistribution HR) [Fine- Gray model]
**Primary Vaccination**		
Partially Vaccinated	42.0% (22.3 - 56.7)	37.7% (27.5 - 46.5)
Fully Vaccinated	51.8% (40.3 - 61.1)	63.7% (55.8 - 70.2)
**Booster Vaccination**		
14 - 60 days after Booster	77.9% (69.2 - 84.2)	54.2% (44.3 - 62.3)
> 60 days after Booster	45.8% (28.7 - 58.9)	69.0% (62.7 - 74.2)

SUPPL. TABLE 4. Vaccine effectiveness before (original VE) and after negative control outcomes calibration. We present the calibrated VE after the original list of negative outcomes (N=43, 'Original Set') and the expanded set of negative outcomes (N=53, 'Expanded Set'). WE present estimates and 95% CI.
NCO = Negative Control Outcomes

		**Calibrated Vaccine Effectiveness**	
	**Vaccine Effectiveness**	**Original Set (N=43 NCO)**	**Expanded Set* (N=53 NCO)**
14 days after 1st Dose	42.0% (22.3 - 56.7)	42.7% (11.2 - 63.0)	43.9% (15.9 - 62.6)
7 days after 2nd dose	51.8% (40.3 - 61.1)	58.2% (43.8 - 68.9)	58.8% (42.6 - 70.5)
14 - 60 days after 3rd dose	77.9% (69.2 - 84.2)	73.5% (61.3 - 81.8)	72.1% (59.3 - 80.8)
60 or more days after 3rd dose	45.8% (28.7 - 58.9)	50.8% (-1.2 - 76.0)	49.7% (2.1 - 74.2)

* = plus low back pain, mild depression, urinalysis, mammography, throat irritation, chest pain, irregular periods, migraine, acetaminophen, amoxicillin and ibuprofen

SUPPL. TABLE 5: Non-COVID outcomes hazard ratio (HR) and estimate vaccine effectiveness (VE) and its confidence interval by periods after primary vaccination (cohort A).

Outcome	Period	N Events	95%CI HR	95%CI Vaccine Effectiveness
All-Cause Hospitalization	no-vax	4463	Ref.	Ref.
All-Cause Hospitalization	V1 0-14D	917	0.68 (0.63 - 0.74)	31.9% (26.0 - 37.4)
All-Cause Hospitalization	V1 14-59D	758	0.90 (0.82 - 0.98)	10.1% (1.8 - 17.7)
All-Cause Hospitalization	V1 60D+	143	0.85 (0.72 - 1.01)	14.7% (-1.3 - 28.2)
All-Cause Hospitalization	V1V2 0-13D	416	0.89 (0.80 - 1.00)	10.7% (0.1 - 20.2)
All-Cause Hospitalization	V1V2 14-59D	836	1.01 (0.93 - 1.09)	-0.6% (-8.6 - 7.5)
All-Cause Hospitalization	V1V2 60-89D	335	0.95 (0.83 - 1.08)	5.3% (-7.2 - 16.7)
All-Cause Hospitalization	V1V2 90-120D	308	1.14 (0.99 - 1.31)	-12.3% (-23.7 - 0.7)
All-Cause Hospitalization	V1V2 120D+	492	1.47 (1.30 - 1.66)	-31.9% (-39.8 - -22.9)
Non-Covid Death	no-vax	2532	Ref.	Ref.
Non-Covid Death	V1 0-14D	32	0.07 (0.05 - 0.10)	93.1% (90.1 - 95.2)
Non-Covid Death	V1 14-59D	168	0.46 (0.39 - 0.55)	53.9% (45.3 - 61.1)
Non-Covid Death	V1 60D+	47	0.40 (0.30 - 0.53)	60.2% (46.6 - 70.3)
Non-Covid Death	V1V2 0-13D	11	0.05 (0.03 - 0.09)	95.2% (91.3 - 97.4)
Non-Covid Death	V1V2 14-59D	147	0.29 (0.24 - 0.34)	71.1% (65.6 - 75.8)
Non-Covid Death	V1V2 60-89D	83	0.33 (0.26 - 0.42)	67.0% (58.4 - 73.9)
Non-Covid Death	V1V2 90-120D	84	0.30 (0.24 - 0.38)	69.7% (61.8 - 76.0)
Non-Covid Death	V1V2 120D+	186	0.44 (0.37 - 0.51)	56.4% (48.6 - 63.1)

SUPPL. TABLE 6: Non-COVID outcomes hazard ratio (HR) and estimate vaccine effectiveness (VE) and its confidence interval by periods after booster vaccination (cohort B).

Outcome	Period	N Events	95%CI HR	95%CI Vaccine Effectiveness
All-Cause Hospitalization	no-vax	1700	Ref.	Ref.
All-Cause Hospitalization	V3 0-14D	306	0.64 (0.55 - 0.74)	35.9% (26.0 - 44.5)
All-Cause Hospitalization	V3 14-28D	186	0.76 (0.63 - 0.92)	23.9% (7.8 - 37.2)
All-Cause Hospitalization	V3 28-60D	223	0.61 (0.52 - 0.73)	38.5% (27.2 - 48.1)
All-Cause Hospitalization	V3 60-120	367	0.82 (0.71 - 0.95)	17.8% (5.4 - 28.6)
All-Cause Hospitalization	V3 120+	252	0.93 (0.78 - 1.11)	6.8% (-10.2 - 22.0)
Non-Covid Death	no-vax	915	Ref.	Ref.
Non-Covid Death	V3 0-14D	7	0.04 (0.02 - 0.09)	95.9% (91.3 - 98.1)
Non-Covid Death	V3 14-28D	11	0.10 (0.05 - 0.18)	90.5% (82.3 - 94.9)
Non-Covid Death	V3 28-60D	35	0.16 (0.11 - 0.22)	84.3% (77.5 - 89.0)
Non-Covid Death	V3 60-120	64	0.21 (0.16 - 0.28)	78.9% (72.2 - 84.0)
Non-Covid Death	V3 120+	95	0.33 (0.26 - 0.42)	66.8% (57.5 - 74.0)

SUPPL. TABLE 7: Proportion of Deaths by COVID-19 diagnosis (COVID-19 death and non-COVID-19 death) with Preceding Hospitalizations for Cohort A and B.

Cohort	Any Hospitalization before non-COVID death	Any Hospitalization before COVID-19 death
Primary Vaccination Cohort (A)	991/3292 (30%)	63/93 (67%)
Booster Cohort (B)	447/1127 (39%)	71/134 (52%)

This means, among the 3,292 non-COVID deaths in Cohort A, only 991 (30%) were hospitalized before dying, while this number was 67% (63/93) among COVID-19 deaths. The same pattern occurred for Cohort B.

SUPPL. TABLE 8: Estimate hazard ratio (HR) and its 95% confidence interval for outcome of health services utilization by vaccination status for cohort A and cohort B

Outcome Name	Vaccination Status	N Events	HR (95% CI)
**Primary Vaccination Scheme (Cohort A)**			
All-cause Outpatient Visit	Unvaccinated	59943	Ref.
	0 - 14 days	15256	0.45 (0.44 - 0.46)
	Partially Vaccinated	22269	1.58 (1.55 - 1.61)
	Fully Vaccinated	5449	0.97 (0.95 - 1.01)
All-cause Telehealth Visit	Unvaccinated	40239	Ref.
	0 - 14 days	18353	0.87 (0.86 - 0.89)
	Partially Vaccinated	10959	1.07 (1.04 - 1.09)
	Fully Vaccinated	9856	1.16 (1.13 - 1.19)
All-cause Home Visit	Unvaccinated	5363	Ref.
	0 - 14 days	1628	0.70 (0.66 - 0.75)
	Partially Vaccinated	2129	1.37 (1.29 - 1.46)
	Fully Vaccinated	1509	0.95 (0.89 - 1.01)
All-cause UCI Visit	Unvaccinated	363	Ref.
	0 - 14 days	42	0.57 (0.39 - 0.84)
	Partially Vaccinated	67	0.66 (0.50 - 0.87)
	Fully Vaccinated	159	0.79 (0.64 - 0.96)
All-cause Inpatient	Unvaccinated	4362	Ref.
	0 - 14 days	899	0.74 (0.68 - 0.80)
	Partially Vaccinated	1110	0.87 (0.81 - 0.94)
	Fully Vaccinated	2198	1.07 (1.01 - 1.14)
**Booster Vaccination Scheme (Cohort B)**			
All-cause Outpatient Visit	Un-boosted	37178	Ref
	0 - 14 days	9790	0.42 (0.41 - 0.43)
	14 - 60 days after booster	5642	0.46 (0.44 - 0.47)
	60 days+ after booster	3204	1.00 (0.95 - 1.05)
All-cause Telehealth Visit	Un-boosted	17482	Ref
	0 - 14 days	8175	0.87 (0.84 - 0.90)
	14 - 60 days after booster	5330	0.87 (0.84 - 0.90)
	60 days+ after booster	3333	1.09 (1.03 - 1.15)
All-cause Home Visit	Un-boosted	3436	Ref
	0 - 14 days	878	0.50 (0.46 - 0.54)
	14 - 60 days after booster	590	0.53 (0.48 - 0.59)
	60 days+ after booster	586	0.85 (0.76 - 0.95)
All-cause UCI Visit	Un-boosted	112	Ref

	0 - 14 days	12	0.50 (0.25 - 1.00)
	14 - 60 days after booster	17	0.36 (0.21 - 0.63)
	60 days+ after booster	57	1.26 (0.84 - 1.87)
All-cause Inpatient	Un-boosted	1667	Ref
	0 - 14 days	302	0.69 (0.60 - 0.80)
	14 - 60 days after booster	409	0.67 (0.59 - 0.76)
	60 days+ after booster	619	0.86 (0.77 - 0.96)

SUPPL. TABLE 9: Baseline Descriptive Characteristics after More Restricted Matching for the Primary Vaccination Cohort (matching on previous influenza vaccine receipt and exclusion of patients hospitalized a month prior to vaccination).

	Unvaccinated	Vaccinated
Number	84308	84308
Age	64.53 (14.98)	64.64 (15.01)
Age		
18-49	14253 (16.9)	14253 (16.9)
50-59	14688 (17.4)	14688 (17.4)
60-69	21316 (25.3)	21316 (25.3)
70-79	21514 (25.5)	21514 (25.5)
80-115	12537 (14.9)	12537 (14.9)
Sex/Gender Female	43221 (51.3)	43221 (51.3)
MEDEA (2001)		
0N	9784 (11.6)	9208 (10.9)
R	12049 (14.3)	12140 (14.4)
U1	13442 (15.9)	14256 (16.9)
U2	12767 (15.1)	13304 (15.8)
U3	12540 (14.9)	12534 (14.9)
U4	12452 (14.8)	12324 (14.6)
U5	11274 (13.4)	10542 (12.5)
Vaccine Dose One		
Pfizer-mRNA-BNT162b		49084 (58.2)
Moderna-mRNA-1273		15602 (18.5)
AZ-ChAdOx1		16552 (19.6)
Jansen-Ad26		3070 (3.6)
NA		0 (0.0)
Vaccine Dose Two		
Pfizer-mRNA-BNT162b		49044 (58.2)
Moderna-mRNA-1273		15356 (18.2)
AZ-ChAdOx1		16079 (19.1)
Jansen-Ad26		0 (0.0)
NA		3829 (4.5)
Time Since Diagnosis (Years)		
0	21114 (25.0)	21114 (25.0)
1	17310 (20.5)	17310 (20.5)
2	16159 (19.2)	16159 (19.2)
3	15083 (17.9)	15083 (17.9)
4	14642 (17.4)	14642 (17.4)

Previous Influenza Vaccination	39077 (46.4)	39077 (46.4)
Charlson Comorbidity Index (CCI)	3.00 [2.00, 8.00]	3.00 [2.00, 5.00]
Metastatic Solid Tumor	11696 (13.9)	11806 (14.0)
Number of Outpatient Visits (category)		
0	9365 (11.1)	8730 (10.4)
1	8043 (9.5)	8417 (10.0)
2	7861 (9.3)	8135 (9.6)
3+	59039 (70.0)	59026 (70.0)

SUPPL. TABLE 10: Baseline Descriptive Characteristics after More Restricted Matching for the Booster Vaccination Cohort. (matching on previous influenza vaccine receipt and exclusion of patients hospitalized a month prior to vaccination). Counts below five have been masked to protect patients privacy.

	Un-boosted	Boosted
Number	46930	46930
Age	69.45 (12.32)	69.45 (12.32)
Age		
18-49	3230 (6.9)	3230 (6.9)
50-59	6988 (14.9)	6988 (14.9)
60-69	12599 (26.8)	12599 (26.8)
70-79	14524 (30.9)	14524 (30.9)
80-115	9589 (20.4)	9589 (20.4)
Sex/Gender Female	22988 (49.0)	22988 (49.0)
MEDEA (2001)		
0N	4283 (9.1)	4176 (8.9)
R	6952 (14.8)	6984 (14.9)
U1	8230 (17.5)	8417 (17.9)
U2	7834 (16.7)	7790 (16.6)
U3	7196 (15.3)	7127 (15.2)
U4	6757 (14.4)	6884 (14.7)
U5	5678 (12.1)	5552 (11.8)
Vaccine Dose Three		
Pfizer-mRNA-BNT162b		11921 (25.4)
Moderna-mRNA-1273		35005 (74.6)
AZ-ChAdOx1		< 5 (0.0)
Jansen-Ad26		0 (0.0)
1st and 2nd Vaccine Combination		
AZ-ChAdOx1-AZ-ChAdOx1	11616 (24.8)	11616 (24.8)
Moderna-mRNA-1273-Moderna-mRNA-1273	3749 (8.0)	3749 (8.0)
Pfizer-mRNA-BNT162b-Pfizer-mRNA-BNT162b	31565 (67.3)	31565 (67.3)
Time Since Diagnosis (Years)		
0	9891 (21.1)	9891 (21.1)
1	9681 (20.6)	9681 (20.6)
2	9447 (20.1)	9447 (20.1)
3	8880 (18.9)	8880 (18.9)
4	9031 (19.2)	9031 (19.2)
Previous Influenza Vaccine	25865 (55.1)	25865 (55.1)

Charlson Comorbidity Index (CCI)	3.00 [2.00, 5.00]	3.00 [2.00, 5.00]
Metastatic Solid Tumor	5141 (11.0)	4888 (10.4)
Number of Outpatient Visits (category)		
0	3904 (8.3)	4198 (8.9)
1	4417 (9.4)	4629 (9.9)
2	4412 (9.4)	4425 (9.4)
3+	34197 (72.9)	33678 (71.8)

SUPPL. TABLE 11: Non-COVID outcomes hazard ratio (HR) and estimate vaccine effectiveness (VE) and its confidence interval by periods for the primary vaccination in the restricted matching cohort (matching on previous influenza vaccine receipt and exclusion of patients hospitalized a month prior to vaccination).

Outcome	Period	N Events	95%CI HR	95%CI Vaccine Effectiveness
All-Cause Hospitalization	no-vax	4202	Ref.	Ref.
All-Cause Hospitalization	V1 0-14D	670	0.75 (0.68 - 0.83)	25.1% (17.2 - 32.3)
All-Cause Hospitalization	V1 14-59D	568	0.94 (0.85 - 1.04)	6.3% (-3.7 - 15.4)
All-Cause Hospitalization	V1 60D+	198	1.14 (0.98 - 1.32)	-12.0% (-24.1 - 2.0)
All-Cause Hospitalization	V1V2 0-13D	329	0.98 (0.87 - 1.12)	1.7% (-10.4 - 13.4)
All-Cause Hospitalization	V1V2 14-59D	607	0.92 (0.84 - 1.01)	8.0% (-1.3 - 16.4)
All-Cause Hospitalization	V1V2 60-89D	300	0.83 (0.73 - 0.95)	17.1% (5.5 - 27.3)
All-Cause Hospitalization	V1V2 90-120D	279	0.73 (0.64 - 0.83)	27.3% (16.9 - 36.4)
All-Cause Hospitalization	V1V2 120D+	909	1.04 (0.95 - 1.13)	-3.8% (-11.7 - 4.6)
Non-Covid Death	no-vax	1737	Ref.	Ref.
Non-Covid Death	V1 0-14D	23	0.08 (0.05 - 0.12)	92.2% (88.1 - 94.9)
Non-Covid Death	V1 14-59D	118	0.52 (0.42 - 0.64)	48.3% (36.5 - 57.9)
Non-Covid Death	V1 60D+	30	0.35 (0.24 - 0.50)	65.0% (49.5 - 75.7)
Non-Covid Death	V1V2 0-13D	10	0.07 (0.04 - 0.14)	92.8% (86.5 - 96.2)
Non-Covid Death	V1V2 14-59D	83	0.24 (0.19 - 0.31)	75.6% (69.3 - 80.6)
Non-Covid Death	V1V2 60-89D	58	0.35 (0.26 - 0.46)	65.2% (54.1 - 73.7)
Non-Covid Death	V1V2 90-120D	47	0.24 (0.18 - 0.32)	76.0% (67.5 - 82.3)
Non-Covid Death	V1V2 120D+	132	0.37 (0.31 - 0.45)	63.0% (55.1 - 69.5)

SUPPL. TABLE 12: Non-COVID outcomes hazard ratio (HR) and estimate vaccine effectiveness (VE) and its confidence interval by periods for the booster vaccination in the restricted matching cohort (matching on previous influenza vaccine receipt and exclusion of patients hospitalized a month prior to vaccination). Counts below five have been masked to protect patients privacy.

Outcome	Period	N Events	95%CI HR	95%CI Vaccine Effectiveness
All-Cause Hospitalization	no-vax	1163	Ref.	Ref.
All-Cause Hospitalization	V3 0-14D	219	0.71 (0.60 - 0.85)	28.9% (15.5 - 40.3)
All-Cause Hospitalization	V3 14-28D	152	0.75 (0.61 - 0.93)	24.9% (7.2 - 39.2)
All-Cause Hospitalization	V3 28-60D	172	0.77 (0.63 - 0.95)	22.6% (5.4 - 36.7)
All-Cause Hospitalization	V3 60-120	257	0.87 (0.73 - 1.03)	13.0% (-3.0 - 26.6)
All-Cause Hospitalization	V3 120+	177	0.95 (0.77 - 1.17)	5.2% (-14.6 - 23.3)
Non-Covid Death	no-vax	560	Ref.	Ref.
Non-Covid Death	V3 0-14D	< 5	0.05 (0.02 - 0.14)	94.8% (85.8 - 98.1)
Non-Covid Death	V3 14-28D	6	0.10 (0.04 - 0.23)	90.2% (77.4 - 95.8)
Non-Covid Death	V3 28-60D	15	0.09 (0.05 - 0.15)	91.4% (85.4 - 94.9)
Non-Covid Death	V3 60-120	34	0.18 (0.13 - 0.27)	81.7% (73.4 - 87.4)
Non-Covid Death	V3 120+	57	0.33 (0.24 - 0.45)	67.1% (54.8 - 76.0)

SUPPL. TABLE 13: COVID-19 vaccine effectiveness and its 95% confidence interval against COVID-19 hospitalization for the primary and booster vaccination for the original cohort and the restricted matching cohort (matching on previous influenza vaccine receipt and exclusion of patients hospitalized a month prior to vaccination).

	Original Cohort	Restricted Matching Cohort
**1st and 2nd Dose Vaccination**		
14 days after 1st Dose	42.0% (22.3 - 56.7)	59.3% (41.0 - 72.0)
7 days after 2nd dose	51.8% (40.3 - 61.1)	64.8% (53.8 - 73.3)
**Booster Dose Vaccination**		
14 - 60 days after 3rd dose	77.9% (69.2 - 84.2)	72.9% (61.2 - 81.0)
60 or more days after 3rd dose	45.8% (28.7 - 58.9)	53.0% (33.1 - 66.9)

SUPPL. TABLE 14: Common data model (CDM) cohort definitions using the Atlas tools from OHDSI. JSON files can be found in the github repository (https://github.com/felippelazar/SIDIAP-CovidVaccineCancer/tree/main/Cohort%20Definitions). To visualized the concept sets included and the full cohort definition do the following: (i) copy cohort JSON text from the github repository (ii) access https://atlas-demo.ohdsi.org/#/cohortdefinitions; (iii) click on create new cohort; (iv) click on 'Export' tab (v) click on 'JSON' tab (vi) paste the JSON text you copied during step (i); (vii) click the 'reload' button; (viii) explore the definition and concept set tabs to visualized the definitions we used. This tutorial was created on 4th August 2023. Future updates of the website may affect the steps used.
Archive Zenodo Files: DOI: 10.5281/zenodo.11237690

**Cohort**
01. Cancer Excluding Non-Melanoma Skin Cancer
02. COVID-19 Testing PCR or Antigen (Excluding Antibody)
03. COVID-19 Vaccine
04. COVID-Diagnosis (Clinical or Laboratorial)
05. COVID-Diagnosis Hospitalized (Clinical or Laboratorial) - 21D bef - allD aft
06. Hospitalization with MV, Tracheostomy or ECMO
07. Cancer Excluding Non-Melanome Skin Cancer - Strict Definition for Melanoma (Excluding Undefined Skin Cancer)
08. Metastatic Cancer
09. COVID-19 Testing PCR or Antigen (Excluding Antibody) - Negative Test
10. COVID-19 Testing PCR or Antigen (Excluding Antibody) - Positive Test
11. COVID-19 Hospitalization (Clinical or Laboratorial) – 21 days before to 3 days after
12. COVID-Diagnosis (Laboratorial Only)
13. COVID-19 Hospitalization (Clinical or Laboratorial) – 14 days before to 3 days after

SUPPL. TABLE 15: Concept IDs used to describe the cancer diagnosis population.
Note: The following concept IDs were used to **describe** and not **define** the cohort. To check cohort definitions, please refer to Suppl. Table 14.

Cancer diagnosis	Cancer Diagnosis Concept IDs
CNS	380055, 4091490, 441233, 433976, 4003693, 433149, 4247822, 438086, 135491, 432848, 441806, 432559, 4246029
bladder	196360, 4095168, 435484, 42709931, 436923, 40650479, 76924, 200054, 195480
breast	137809, 4162253, 133711, 436353, 440956, 441515, 432263, 4091464, 432845, 135489, 4092513, 4187850, 441513, 4091469, 4247348, 36684817, 4003684, 4188545, 36684820, 4158563, 4187851
cervix-uterus	196359, 441805, 4092515, 436358, 4162860, 45770892, 198082, 201801, 4247842, 192847, 4095748, 196047
colorectal	197500, 74582, 436635, 4247719, 438699, 432837, 441800, 432257, 438979, 79740, 437798, 438090, 433143, 435754, 443391, 443381, 443382, 443384
endometrium	4247238
esophagus	26638, 193138, 437805, 432260, 25748, 4094863
gastric	196044, 438089, 432838, 197803, 193422, 435751, 434292, 437224, 192255
head-neck	435190, 440044, 435474, 434577, 26052, 260336, 261514, 439746, 22839, 259755, 436643, 438367, 439404, 437498, 256633, 135750, 140955, 4156114, 436043, 136915, 444224, 132565, 134579, 433704, 440335, 133969, 133710, 134290, 4155171, 140950, 254282, 435478, 4001170, 438694, 138074, 137219, 434587, 438982, 437220, 440344, 255192, 436042, 261808, 138351, 132832, 432833, 31509, 438691, 28083, 434285, 4002498, 438360, 440047, 440345, 439739, 434289, 433709, 137800, 253977, 140046, 136639, 132258
kidney	198985
leukemia	4082311, 140352, 134305, 134603, 317510, 141816, 135496, 132853, 4133599, 133438, 138708, 140666, 132572, 4038845, 133154, 4189938, 140057, 4002497, 40483761, 134596, 135766, 4079686, 132850, 4079282, 4137687, 138099, 135762, 321526, 443719, 4173974, 4173963, 133158, 134597, 40482847, 4079683, 760936, 4002496, 4139358, 40481524
liver-biliary	197806, 441225, 40486896, 194589, 198695, 4095432, 4001171, 4246127, 201519
lung	4311499, 261236, 4157454, 258375, 257503, 256646, 4334322, 252840
lymphoma	4147164, 40487528, 4038838, 4300704, 4147411, 132841, 440058, 194878, 438698, 4003830, 200349, 434592, 435753, 320347, 200343, 4003833, 4001328, 40482893, 200338, 192560, 4002356, 4097560, 40481901, 4002357, 4001329, 315481, 4184976, 435492, 4041800, 434881, 4212994, 4091768, 4029188, 4003188, 4082487, 4299149, 441521, 44808122, 40481357, 40486171, 40481522, 4038839, 198374, 40479608, 45765770, 4097561, 40488896, 198088, 4301668, 4082627, 4287493, 196055, 40483374, 195195, 193428, 4038841, 35610325, 4301666, 439293
melanoma	141232, 4149851, 133713, 133419, 4244488, 434590, 4244051
myeloma	437233
neuroendocrine	37018875, 25486, 46271402, 37017321, 37018934, 40482859

other	198104, 4162859, 4162115, 80045, 436348, 40481902, 4033318, 436352, 444203, 438693, 432844, 321234, 435487, 316644, 378696, 4002340, 436926, 134295, 78093, 4111917, 4033891, 4032870, 432558, 436344, 36716501, 438080, 441223, 438692, 377811, 438095, 374874, 4040379, 26361, 373151, 24296, 139753, 201517, 133420, 4247336, 193418, 4246808, 372567, 436922, 375490, 4181477, 442134, 432264, 4003674, 259748, 27235, 200963, 432254, 434588, 195483, 438370, 4003027, 197507, 79749, 440339, 197799, 198092, 4089665, 433975, 4002343, 442122, 4094260, 4246137, 4110889, 4003175, 4095892, 4097283, 4097284, 4094262, 4091621, 4114198, 4003179, 198091, 439738, 4247836, 192836, 435752, 4311480, 436913, 199747, 438368, 443380, 195197, 441802, 196048, 4003029, 437501, 197225
ovary	200051, 200052
pancreas	199754, 440649, 192261, 432843, 434293, 433423
prostate	200962
sarcomas	76914, 436640, 4246141, 379756, 75488, 81237, 438977, 4246802, 198988, 435493, 434880, 4089777, 376647, 4095018, 4092235, 4092358, 378081
testis	433716, 436054, 79758, 193719, 80340, 4003028
thyroid	133424
undefined	139750, 439392, 136917, 4157456, 133974, 133147, 443392, 4312685, 4095598, 4179720, 435755, 201518, 4162994, 442131, 201238, 76349, 196049, 4003834, 4091486, 195482, 435485, 196645, 4312698, 432262, 196051, 81239, 4114221, 4197474, 4197475, 45757107
hematological	4082311, 140352, 134305, 134603, 317510, 141816, 135496, 132853, 4133599, 133438, 138708, 140666, 132572, 4038845, 133154, 4189938, 140057, 4002497, 40483761, 134596, 135766, 4079686, 132850, 4079282, 4137687, 138099, 135762, 321526, 443719, 4173974, 4173963, 133158, 134597, 40482847, 4079683, 760936, 4002496, 4139358, 40481524, 4147164, 40487528, 4038838, 4300704, 4147411, 132841, 440058, 194878, 438698, 4003830, 200349, 434592, 435753, 320347, 200343, 4003833, 4001328, 40482893, 200338, 192560, 4002356, 4097560, 40481901, 4002357, 4001329, 315481, 4184976, 435492, 4041800, 434881, 4212994, 4091768, 4029188, 4003188, 4082487, 4299149, 441521, 44808122, 40481357, 40486171, 40481522, 4038839, 198374, 40479608, 45765770, 4097561, 40488896, 198088, 4301668, 4082627, 4287493, 196055, 40483374, 195195, 193428, 4038841, 35610325, 4301666, 439293, 437233, 4040379, 4003834

SUPPL. TABLE 16: Charlson Comorbidity Index Ancestor Concept IDs

Charlson Comorbidities	Ancestor Concept IDs
AIDS	439727
Any Malignancy	443392
Cerebrovascular Disease	381591, 434056
Chronic Pulmonary Disease	4063381
Congestive Heart Failure	316139
Dementia	4182210
Diabetes Mild	201820
Diabetes With Complications	443767, 442793
Hemiplegia Paraplegia	192606, 374022
Metastatic Solid Tumor	432851
Mild Liver Disease	4064161, 4212540
Moderate Severe Liver Disease	4245975, 4029488, 192680, 24966
Myocardial Infarction	4329847
Peptic Ulcer Disease	4247120
Peripheral Artery Disease	321052
Renal Disease	4030518
Rheumatologic Disease	257628, 134442, 80800, 80809, 256197, 255348

SUPPL. TABLE 17: Negative Outcomes Names and Concept ID
** = Expanded Set of Negative Outcomes (Sensitivity Analysis)

ConceptId	OutcomeName	ConceptID	OutcomeName
75860	Constipation	196456	Gallstone
197304	Ulcer of lower extremity	135333	Pressure ulcer
42709838	Cellulitis of lower limb	4285898	Polyp of colon
436659	Iron deficiency anemia	374375	Impacted cerumen
4155902	Wax in ear canal	443419	Laceration - injury
138825	Actinic keratosis	4053604	Open wound of lower leg
375545	Cataract	4060207	Vulval irritation
377889	Hearing loss	376707	Acute conjunctivitis
140673	Hypothyroidism	4058568	Vaginal irritation
4026112	Rectal hemorrhage	4016155	Prostatism
4169905	Foot pain	194133	Low back pain**
197672	Urinary incontinence	4149320	Mild depression**
4317977	Bilateral cataracts	4099313	Urinalysis**
436070	Vitamin D deficiency	4324693	Mammography**
4112752	Basal cell carcinoma of skin	4038048	Throat irritation**
195562	Hemorrhoids	77670	Chest pain**
141932	Senile hyperkeratosis	196168	Irregular periods**
4217260	Intraocular pressure left eye	318736	Migraine**
4038030	Hearing difficulty	1125315	Acetaminophen**
437541	Glaucoma	1177480	Ibuprofen**
380731	Otitis externa	1713332	Amoxicilline**
4195039	Osteopenia		
4036620	Dry eyes		
198803	Benign prostatic hyperplasia		
378425	Blepharitis		
74719	Ulcer of foot		
4111921	Squamous cell carcinoma of skin		
138384	Acquired hypothyroidism		
374028	Age related macular degeneration		
44783954	Acid reflux		
4155040	Laceration of lower leg		
4288544	Inguinal hernia		
46287159	Traumatic wound		
