# Supplementary material for: Effectiveness of COVID-19 vaccines against severe COVID-19 among patients with cancer in Catalonia, Spain
Source: Nat Commun. 2024 Jun 19;15:5088. doi: 10.1038/s41467-024-49285-y (PMC11187152; doi:10.1038/s41467-024-49285-y)
Supplement: Supplementary file 3 — Reporting Summary [file 41467_2024_49285_MOESM3_ESM.pdf]

nature portfolio

Corresponding author(s): Otavio T. Ranzani, Talita Duarte-Salles

Last updated by author(s): 05/21/2024

Reporting Summary

Nature Portfolio wishes to improve the reproducibility of the work that we publish. This form provides structure for consistency and transparency in reporting. For further information on Nature Portfolio policies, see our Editorial Policies and the Editorial Policy Checklist.

Statistics

For all statistical analyses, confirm that the following items are present in the figure legend, table legend, main text, or Methods section.

n/a	Confirmed	
☐	☒	The exact sample size (*n*) for each experimental group/condition, given as a discrete number and unit of measurement
☒	☐	A statement on whether measurements were taken from distinct samples or whether the same sample was measured repeatedly
☒	☐	The statistical test(s) used AND whether they are one- or two-sided<br>*Only common tests should be described solely by name; describe more complex techniques in the Methods section.*
☐	☒	A description of all covariates tested
☒	☐	A description of any assumptions or corrections, such as tests of normality and adjustment for multiple comparisons
☐	☒	A full description of the statistical parameters including central tendency (e.g. means) or other basic estimates (e.g. regression coefficient) AND variation (e.g. standard deviation) or associated estimates of uncertainty (e.g. confidence intervals)
☐	☒	For null hypothesis testing, the test statistic (e.g. *F*, *t*, *r*) with confidence intervals, effect sizes, degrees of freedom and *P* value noted<br>*Give P values as exact values whenever suitable.*
☒	☐	For Bayesian analysis, information on the choice of priors and Markov chain Monte Carlo settings
☒	☐	For hierarchical and complex designs, identification of the appropriate level for tests and full reporting of outcomes
☒	☐	Estimates of effect sizes (e.g. Cohen's *d*, Pearson's *r*), indicating how they were calculated

Our web collection on statistics for biologists contains articles on many of the points above.

Software and code

Policy information about availability of computer code

Data collection	Individual-level routinely-collected primary care data were extracted from the Information System for Research in Primary Care (SIDIAP; www.sidiap.org) database, which captures patient records from approximately 80% of the Catalan population
Data analysis	We performed all analyses in R version 3.6.0 (R Foundation for Statistical Computing, Vienna, Austria). R scripts were made available to ensure the reproducibility of results and in accordance with good research practice (https://github.com/felippelazar/SIDIAP-CovVaxCan).

For manuscripts utilizing custom algorithms or software that are central to the research but not yet described in published literature, software must be made available to editors and reviewers. We strongly encourage code deposition in a community repository (e.g. GitHub). See the Nature Portfolio guidelines for submitting code & software for further information.

Data

Policy information about availability of data

All manuscripts must include a data availability statement. This statement should provide the following information, where applicable:

- Accession codes, unique identifiers, or web links for publicly available datasets
- A description of any restrictions on data availability
- For clinical datasets or third party data, please ensure that the statement adheres to our policy

In accordance with the current European and national law, the data used in this study are only available for the researchers participating in this study. Thus, we are not allowed to distribute or make publicly available the data to other parties. However, researchers from public institutions can request data from SIDIAP if they

comply with certain requirements. Further information is available online (https://www.sidiap.org/index.php/menu-solicitudesen/application-proccedure) or by contacting SIDIAP (sidiap@idiapjgol.org).

Research involving human participants, their data, or biological material

Policy information about studies with human participants or human data. See also policy information about sex, gender (identity/presentation), and sexual orientation and race, ethnicity and racism.

Reporting on sex and gender	We used sex in this manuscript, because we have data extracted from administrative databases and sex was determined as in the birth certificate/national card identification. We provided sex-specific estimates in sub-group analysis.
Reporting on race, ethnicity, or other socially relevant groupings	We have not reported race and/or ethnicity as this data is not routinely collected in the database.
Population characteristics	We included 92,372 pairs of vaccinated and control patients with cancer in Cohort A and 54,267 in Cohort B, which is a subset of Cohort A. The mean age was 64 years (SD 15.12) for Cohort A and 69 years (SD 12.42) for Cohort B. Both cohorts had similar distributions of sex (male 49%, female 51%) and the most common cancer diagnosis (breast 17%, prostate 13%, colorrectal 13%, lung 5%). The majority of patients had a recent cancer diagnosis (≤ 1 year: 21%, 1-2 years: 20%, 2-3 years: 19%).
Recruitment	We conducted a population-based cohort of adult population, so no expected risk for selection bias.
Ethics oversight	The current work was approved by the Clinical Research Ethics Committee of IDIAPJGol (project code 23/023-EOm).

Note that full information on the approval of the study protocol must also be provided in the manuscript.

Field-specific reporting

Please select the one below that is the best fit for your research. If you are not sure, read the appropriate sections before making your selection.

☒ Life sciences ☐ Behavioural & social sciences ☐ Ecological, evolutionary & environmental sciences

For a reference copy of the document with all sections, see nature.com/documents/nr-reporting-summary-flat.pdf

Life sciences study design

All studies must disclose on these points even when the disclosure is negative.

Sample size	We did not perform a formal power analysis for this study. We aimed for a population-based cohort of whole adult cancer population in Catalonia. Being a population-based study, including all population of the region, we did not expect any problem with power.
Data exclusions	A detailed description of all inclusion and exclusion criteria is described in supplementary flowcharts (Suppl. Fig. 1 and Suppl. Fig. 2). Overall, we included (i) adult patients; (ii) with a cancer diagnosis (up to five years from date of vaccination); (iii) without any previous COVID-19 infection; (iv) excluding nurse home residents; (v) and excluding patients that moved-out from Catalonia.
Replication	We did not perform any replication experiment in this study. However, we did were several sensitivity analyses, as in epidemiological methods, using the same population, but testing different exposures, outcomes, which produced similar findings to the primary analysis.
Randomization	This is an observational study, so no randomization occurred. We used a sequential adjustment approach to reduce bias and confounding.
Blinding	This was a retrospective study, and blinding was done when possible. Thus, we designed the protocol and statistical analysis plan before extracting any data. This study did not use any kind of training, test and validation subsets.

Reporting for specific materials, systems and methods

We require information from authors about some types of materials, experimental systems and methods used in many studies. Here, indicate whether each material, system or method listed is relevant to your study. If you are not sure if a list item applies to your research, read the appropriate section before selecting a response.

Materials & experimental systems

n/a	Involved in the study	
☒	☐	Antibodies
☒	☐	Eukaryotic cell lines
☒	☐	Palaeontology and archaeology
☒	☐	Animals and other organisms
☒	☐	Clinical data
☒	☐	Dual use research of concern
☒	☐	Plants

Methods

n/a	Involved in the study	
☒	☐	ChIP-seq
☒	☐	Flow cytometry
☒	☐	MRI-based neuroimaging

Plants

Seed stocks	Not applicable.
Novel plant genotypes	Not applicable.
Authentication	Not applicable.
